# Salactin, a dynamically unstable actin homolog in Haloarchaea

**DOI:** 10.1128/mbio.02272-23

**Published:** 2023-11-15

**Authors:** Jenny Zheng, John Mallon, Alex Lammers, Theopi Rados, Thomas Litschel, Edmund R. R. Moody, Diego A. Ramirez-Diaz, Amy Schmid, Tom A. Williams, Alexandre W. Bisson-Filho, Ethan Garner

**Affiliations:** 1 Department of Molecular and Cellular Biology, Harvard University, Cambridge, Massachusetts, USA; 2 Department of Biology, Rosenstiel Basic Medical Science Research Center, Brandeis University, Waltham, Massachusetts, USA; 3 Physiology Course, Marine Biological Laboratory, Woods Hole, Massachusetts, USA; 4 Department of Biomedical Engineering, The Biological Design Center, Boston University, Boston, Massachusetts, USA; 5 The Wyss Institute for Biologically Inspired Engineering, Harvard University, Boston, Massachusetts, USA; 6 John A. Paulson School of Engineering and Applied Sciences, Harvard University, Cambridge, Massachusetts, USA; 7 School of Earth Sciences, University of Bristol, Bristol, United Kingdom; 8 Department of Biology, Duke University, Durham, North Carolina, USA; 9 Center for Genomics and Computational Biology, Duke University, Durham, North Carolina, USA; 10 School of Biological Sciences, University of Bristol, Bristol, United Kingdom; Institut Pasteur, Paris, France

**Keywords:** actin, archaea, cytoskeleton, DNA segregation, dynamic instability

## Abstract

Across the domains of life, actin homologs are integral components of many essential processes, such as DNA segregation, cell division, and cell shape determination. Archaeal genomes, like those of bacteria and eukaryotes, also encode actin homologs, but much less is known about these proteins’ *in vivo* dynamics and cellular functions. We identified and characterized the function and dynamics of Salactin, an actin homolog in the hypersaline archaeon *Halobacterium salinarum*. Live-cell time-lapse imaging revealed that Salactin forms dynamically unstable filaments that grow and shrink out of the cell poles. Like other dynamically unstable polymers, Salactin monomers are added at the growing filament end, and its ATP-bound critical concentration is substantially lower than the ADP-bound form. When *H. salinarum’s* chromosomal copy number becomes limiting under low-phosphate growth conditions, cells lacking Salactin show perturbed DNA distributions. Taken together, we propose that Salactin is part of a previously unknown chromosomal segregation apparatus required during low-ploidy conditions.

**IMPORTANCE:**

Protein filaments play important roles in many biological processes. We discovered an actin homolog in halophilic archaea, which we call Salactin. Just like the filaments that segregate DNA in eukaryotes, Salactin grows out of the cell poles towards the middle, and then quickly depolymerizes, a behavior known as dynamic instability. Furthermore, we see that Salactin affects the distribution of DNA in daughter cells when cells are grown in low-phosphate media, suggesting Salactin filaments might be involved in segregating DNA when the cell has only a few copies of the chromosome.

## INTRODUCTION

Actin and its homologs are present in all domains of life and are involved in various processes, such as cell motility, division, shape determination, and DNA segregation (1–4). Eukaryotic actin is critical for the motility, division, and shape of eukaryotic cells (5, 6). Bacteria also contain actin homologs that are involved in a variety of functions: (i) MreB creates and maintains rod shape (7), (ii) MamK positions magnetosomes along the cell length (8, 9), (iii) FtsA is a central component of the division machinery (10), and (iv) plasmid-encoded actin proteins such as AlfA and ParM ensure low-copy plasmid inheritance (11–15). Phylogenomics identified several actin and tubulin homologs in archaea (16–19). Two archaeal tubulin homolog families (FtsZ and CetZ) have been visualized in the haloarchaeon *Haloferax volcanii* and determined to be involved in division and cell shape, respectively (20, 21).

Comparatively, the functions of archaeal actins have been far less studied: we still lack an understanding of the *in vivo* dynamics or function of any archaeal actin. Various studies have made progress in our understanding: Crenactin’s (from Crenarchaeota) localization and correlation with cell shape suggest that it could be involved in cell shape formation (22–24). Bioinformatics identified multiple actins in the Thaumarchaeota/Aigarchaeota/Crenarchaeota/Korarchaeota (TACK) and Asgard families with high sequence similarity to eukaryotic actin (17, 19, 25–30), and the Asgard genomes also encode multiple eukaryotic-like actin-modulating proteins (29, 31–35). Accordingly, cryo-electron microscopy of Loki Asgard archaea revealed long eukaryotic-like actin filaments enriched within cellular protrusions (36).

Motivated by previous bioinformatic analyses suggesting Haloarchaea actin homologs (16), we searched for actin homologs in the archaeal model *Halobacterium salinarum*. Here, we identified and characterized an actin homolog in *H. salinarum,* which we named Salactin.

## RESULTS

### Identification of Salactin

To search for putative actin fold proteins suggested by Makarova and colleagues (16), we used *Escherichia coli’s* MreB protein sequence as input to JackHMMER, a statistical tool based on hidden Markov models (HMM) (37). Limiting the results to archaeal proteins, we initially obtained 273 candidates across multiple phyla, but most were annotated as HSP70/DnaK (245 hits) or MreB (28 hits). However, a second JackHHMER iteration revealed an additional 86 candidates, 37 of which originated from HSP70 sequences from the first iteration and aligned to an unknown sub-domain. Further inspection showed that most of them had a predicted actin fold structure. Of these*,* we identified a conserved actin homolog in *H. salinarum* (GenBank AAG18772.1)*,* which we named Salactin.

**Fig 1:**
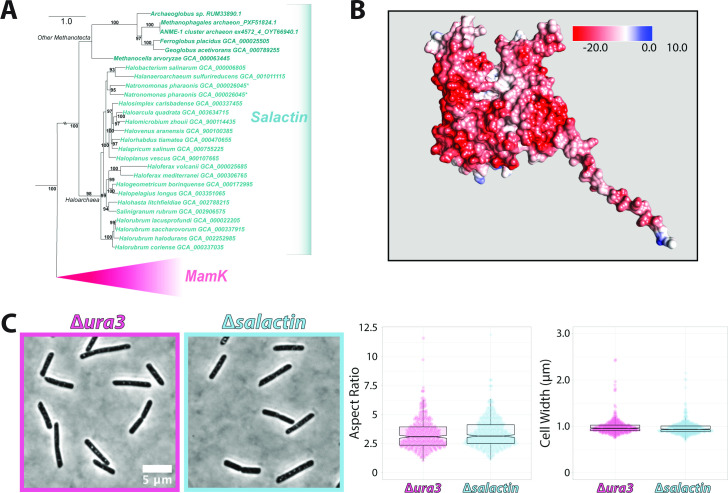
(**A**) Tree of the representative species in the Methanotecta clade in the Euryarchaeota phylum with MamK, a closely related homolog of Salactin. Phylogeny of *salactin* homologs in Haloarchaea and other Methanotecta; the topology indicates that *salactin* was likely already present in the common ancestor of Methanotecta. Branch supports are ultrafast bootstraps (39); only supports >90% are indicated. Branch length is proportional to the expected number of substitutions per site (indicated by the scale bar). All alignments and trees are available in File S1. (**B**) Alphafold2-predicted structure of Salactin colored by its surface electrostatic potential (available from Uniprot, ID: Q9HSN1). (**C**) Phase-contrast images of ∆*ura3* parent strain (pink) and ∆*salactin* (blue) *H. salinarum* cells in rich media (CM + URA) (left) showing that both exhibit the same rod shape. Both images are on the same scale, and the scale bar on the left applies to both panels. Violin plot of the aspect ratio (mid) and width (right) of ∆*salactin* cells and ∆*ura3* cells demonstrates no statistically significant difference between strains (width *P* = 0.0138, aspect ratio *P* = 0.4093). Data were taken across three biological replicates with a total *N* = 375 and 493 for ∆*ura3 and* ∆*salactin* cells*,* respectively.

Phylogenetic analysis of *salactin* (Fig. 1A; Fig. S1) indicated that the gene is broadly conserved across the Haloarchaea, with closely related homologs also present in the other lineages of Methanotecta (the euryarchaeotal clade comprising Haloarchaea, Methanomicrobia, Methanocellales, Methanophagales, and Archaeoglobi) (38). The gene is generally present in a single copy but appears to be duplicated in *Natromonas*. The conservation of Salactin across Methanotecta and the broad agreement between a phylogeny of Salactin homologs and the species phylogeny (Fig. 1A) suggest that *salactin* was already present in the common ancestor of Methanotecta. This result suggests that the function of Salactin is unlikely to be specific to Haloarchaea in that it originated prior to the evolution of halophiles from their methanogenic ancestors. The subfamily of actin-fold proteins most closely related to Salactin appears to be MamK, a result that holds both in a focused tree of Salactin and its most closely related actin homologs (Fig. 1A) as well as a broader phylogenetic analysis of actin family diversity (Fig. S1A through E).

Salactin’s sequence contains the characteristic nucleotide binding motifs found within HSP70/actin fold proteins (40). Structural predictions by AlphaFold2 (41) suggested a canonical actin fold (Uniprot ID: Q9HSN1) with a long, highly charged disordered tail on the N-terminus (Fig. 1B). We conducted backbone alignments of the Alphafold-predicted structure of Salactin to the structures of other polymerizing actin homologs. Of these, the predicted Salactin structure had the best fits (lowest root mean square deviations [RSMDs]) to MreB and MamK, both of which also had the highest percent identity to Salactin at the amino acid level (Fig. S1F; Table S1).

Given many bacterial actins are involved in cell shape, we first tested if Salactin functioned as an MreB, actin homologs required for the establishment and maintenance of rod shape in many bacteria (7, 42). To determine if Salactin has a similar function as MreB and is required for *H. salinarum’s* rod shape, we created a strain where *salactin* was deleted in a ∆*ura3* background (∆*salactin,* ∆*ura3,* further referred to as ∆*salactin*) and a ∆*ura3* strain that we used throughout this work as our control. Because *H. salinarum* is highly polyploid, we performed whole-genome sequencing and determined that ∆*salactin* cells are devoid of *salactin* sequences and, therefore, deleted from all chromosomal copies. Furthermore, we confirmed that no second site suppressor mutations were detected in comparison to the ∆*ura3* sequences, thereby ruling out *salactin* essentiality in rich media (File S3).

Single-cell image analysis from phase-contrast microscopy revealed that ∆*salactin* cells in the exponential phase have rod shapes that are indistinguishable from ∆*ura3* cells, with no significant difference in width, aspect ratio, area, length, or circularity relative to ∆*ura3* cells (width *P* = 0.0138, aspect ratio *P* = 0.4093, area *P* = 0.2721, length *P* = 0.9198, circularity *P* = 0.3924) (Fig. 1C; Fig. S2). As deletion of *salactin* has no effect on the rod shape of *H. salinarum*, these experiments suggested that *H. salinarum*’s Salactin does not functionally act similar to an MreB, either the MreBs that guide the circumferential cell wall synthesis to create and maintain rod shape (7, 43) or those required for the widening and division of nematode attached bacteria (44).

### Salactin filaments display dynamic instability *in vivo*

**Fig 2:**
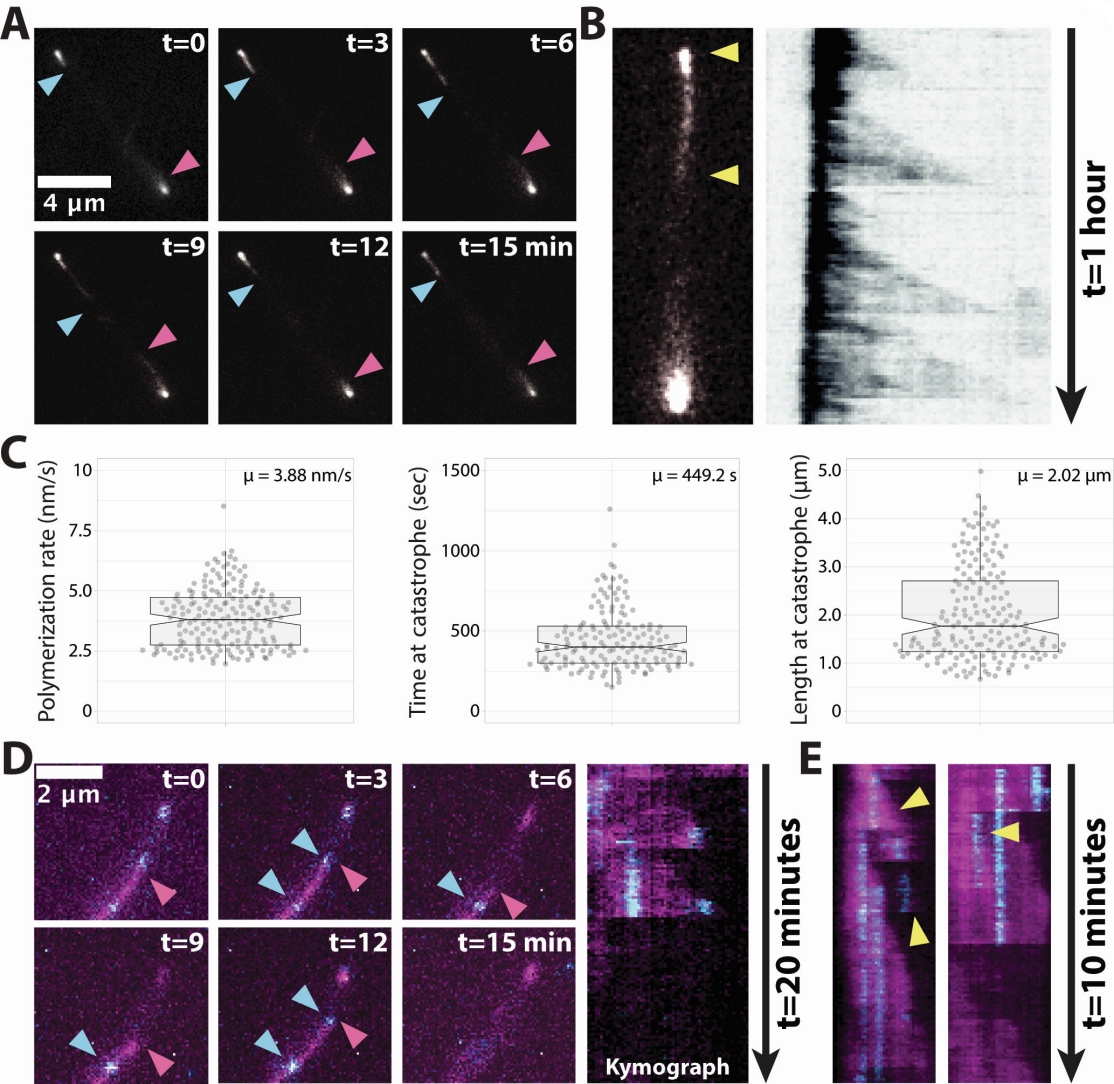
Characterization of *in vivo* Salactin dynamics. (**A**) A montage of Salactin-msfGFP from a *H. salinarum* cell (strain hsJZ52). The two arrowheads indicate each end of the filament. A gamma filter correction of 1 was applied to improve visibility. Images are on the same scale, and the scale bar on the first panel applies to all panels. (**B**) A representative cell (left) is used to create a kymograph (right). Yellow arrowheads indicate the region used for drawing the corresponding kymograph. The kymograph was inverted to improve visibility. (**C**) Violin plot of measured *in vivo* polymerization rates (left, *N* = 184), time until catastrophe (mid, *N* = 152), and length at catastrophe (right, *N* = 181) obtained from analysis of 50 kymographs. (**D**) A montage of a speckle labeled Salactin*-*HaloTag filament in a *H. salinarum* cell (strain hsJZ86) (left). The entire Salactin*-*HaloTag polymer was labeled with JF505 (magenta) and also sparsely labeled with JF549 to generate speckles (cyan). Red arrowhead indicates the filament end, and blue arrowhead indicates a single molecule. Kymograph for the filament trace showing that monomers remain stationary within the growing filament (right). Images are on the same scale, and the scale bar on the first panel applies to all panels. (**E**) Two example kymographs showing multiple triangles (indicated by yellow arrowheads) arising from diffraction-limited filaments, indicating these structures may be composed of multiple filaments.

To gain further insight into Salactin’s function, we examined its localization and dynamics *in vivo*. We created constructs by fusing Salactin to either monomeric superfolder GFP (msfGFP) or HaloTag that were driven by a constitutive, strong ribosomal promoter (*prpa*) on a high-copy-number plasmid. By expressing Salactin-msfGFP ectopically (strain hsJZ52) from a plasmid along with the native copy of *salactin*, we visualized Salactin-msfGFP’s localization and dynamics using time-lapse fluorescence microscopy. This showed that cells had bright foci at their poles (86% with bipolar foci and 14% of cells with unipolar foci). Surprisingly, these movies revealed that Salactin polymers grew out of the polar foci toward the midcell, then would suddenly depolymerize, with filaments rapidly shrinking back to the poles (Fig. 2A; Video S1; Table S2), a behavior observed in 74.55% of cells. The stochastic switching between steady elongation and rapid depolymerization is known as dynamic instability (45). Dynamic instability can be seen in kymographs created with a line from the pole to the mid-cell, where dynamic instability creates right triangles in the kymograph (Fig. 2B): the hypotenuse is the slow phase of polymerization, and the adjacent side arises from rapid depolymerization (46). We observed that this behavior of Salactin would occur multiple times from one pole during a 1-hour observation, as shown by the repeating triangles in the kymographs. Analysis of 50 kymographs indicated Salactin-msfGFP filaments grow at a rate of 3.88 nm/s (Fig. 2C), which (assuming a double-stranded filament and a dimer subunit rise of 5 nm) would cause the filament to grow by 1.5 monomers each second. In contrast, all filament catastrophes occurred within one frame, a rate so fast we could not measure the actual depolymerization rate. By examining the longest filament that depolymerized within a single frame, we could estimate Salactin-msfGFP’s minimal depolymerization rate to be 600 nm/s or 240 monomers per second. The distribution of filament lengths and time before catastrophe had a mean of 2.02 µm and 449.2 s, respectively (Fig. 2C). The distribution of the time before catastrophe appears to have an exponential tail (*R*^2^ = 0.9643 and 0.9335 for exponential fit and linear fit to log frequency, respectively) (Fig. S3), in line with the expected distribution of a broad class of models of dynamic instability (47).

We note that these measurements may not be exactly the same as untagged Salactin filaments inside the cell, as (similar to many other biological polymers) fluorescent fusions to Salactin appear to affect its polymerization; when Salactin was fused to HaloTag and expressed ectopically from a plasmid in the presence of the native copy of *salactin* (strain hsJZ86), dynamically unstable filaments were observed in 61.18% of cells (Fig. 2D; Table S2; Videos S2 and S3; Fig. S4A). However, cells expressing Salactin-HaloTag as the sole copy (strain hsJZ106) showed a considerably reduced fraction of cells showing dynamic instability (38.76%) (Table S2, Video S4, and Fig. S4B). Likewise, cells expressing Salactin-msfGFP as the sole copy (strain hsJZ95) in the cell showed filaments in only 2.4% of cells, and none of these filaments showed any dynamics (Table S3; Video S5; Fig. S4C). Also, Salactin-HaloTag fusions expressed as the only copy in the ∆*salactin* background (strain hsJZ106) yielded a different polymerization rate than when Salactin-HaloTag was expressed ectopically in wild-type cells in addition to the native copy (strain hsJZ86) (Fig. S4D). We conducted RNAseq to determine the levels of Salactin in the ectopic *prpa salactin-msfGFP* strain, finding this strain had 1.7 times the amount of Salactin RNA as wild-type cells (Fig. S4E), a value which should neither affect the on or off-rate constants of the polymer nor the presence or absence of dynamic instability (45, 48–52).

### Salactin monomers are added at the growing filament end

To determine where Salactin monomers are added into growing filaments, we “speckle labeled” Salactin-HaloTag filaments by incubating cells with two Janelia Fluor dyes, JF549 at very low levels (to speckle filaments) and JF505 at much higher levels (to label the rest of the filament). Timelapse microscopy of these cells revealed that the JF549 speckles were stationary within growing Salactin filaments: speckles appeared as filaments grew and remained in the same place until they disappeared when the filament depolymerized (Fig. 2D; Video S2). This demonstrates that new monomers are added to the end of the growing filament rather than being added at the filament ends at the cell poles. In addition, kymographs showed multiple depolymerization events within what appeared to be a single filament, suggesting that some of the diffraction-limited polymers may contain multiple filaments (Fig. 2E), similar to what was observed with ParM filaments in *E. coli* (53, 54). Taken together, these observations demonstrate that Salactin forms dynamically unstable filaments inside *H. salinarum*.

### Salactin polymerization *in vitro*

**Fig 3:**
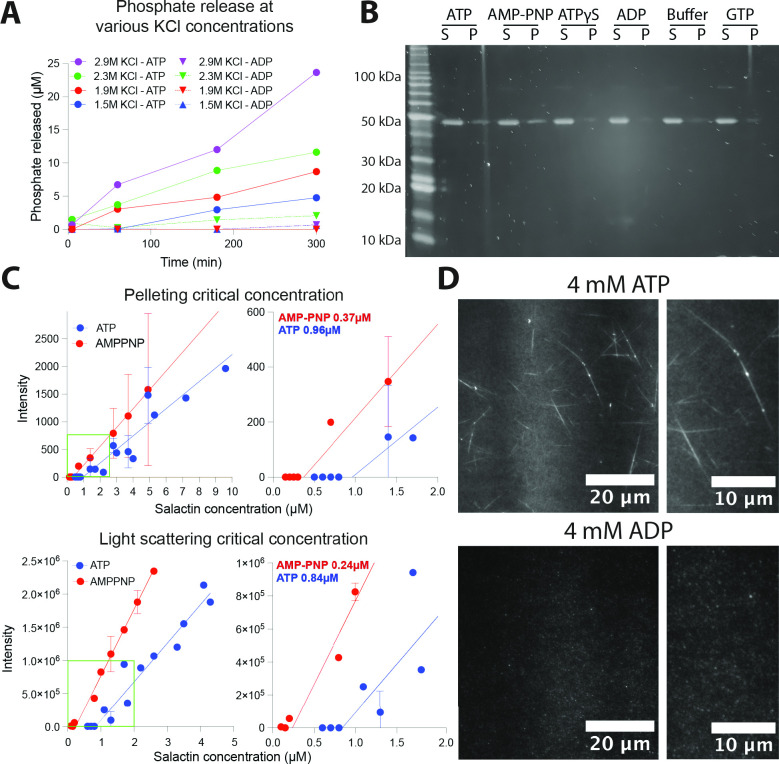
*In vitro* polymerization of Salactin. All assays were done with 2.9 M KCl unless otherwise noted. (**A**) Malachite green assay using 4 µM Salactin in different salt conditions (1.5, 1.9, 2.3, and 2.9 M). The higher ATPase activity implies polymerization is favored at higher salt concentrations. (**B**) Salactin polymerization only occurs in the presence of ATP and AMP-PNP. Salactin polymerization was assayed by pelleting, using 2.5 mM ATP, ATP analogs, ADP, GTP, or buffer alone. Gels were stained with SYPRO Orange. S, supernatant; P, pellet. (**C**) Critical concentrations of Salactin determined by pelleting (top) and light scattering (bottom). (**D**) Polymers of Salactin mixed with cy3B-conjugated Salactin-GSKCK are seen with TIRF microscopy in the presence of ATP but not ADP. Right panels are zoomed-in images of the left panel.

To further understand Salactin, we purified Salactin to examine its polymerization *in vitro* (Fig. S5). Given *H. salinarum* is a halophile, we first optimized buffer KCl concentration using a malachite green assay to indirectly measure polymerization. This revealed that Salactin’s ATPase activity increased with increasing salt (Fig. 3A; Fig. S6), an unsurprising result given the cytoplasm of *H. salinarum* contains ~4.5 M KCl (55, 56). Given that the solubility of KCl is ~3.55 M at room temperature (57), we used the highest KCl concentration (2.9 M) that yielded (i) the highest ATPase activity and (ii) reproducible results without precipitation in our assays.

We first conducted pelleting assays to quantify Salactin’s *in vitro* polymerization. This revealed that 4.9 µM of Salactin polymerizes in the presence of ATP and AMP-PNP but not in the presence of ADP, GTP, ATPγS, or buffer alone (Fig. 3B). Next, we measured the critical concentrations of Salactin in the presence of different nucleotides, as dynamic instability arises from a substantial difference in the dissociation constants of the ATP and ADP-bound monomers for filament ends (52, 58). We measured Salactin’s ATP and AMP-PNP critical concentrations using both pelleting and right-angle light scattering. Both assays gave similar values: pelleting yielded a critical concentration of 0.96 µM for ATP and 0.37 µM for AMP-PNP, and light scattering yielded 0.84 µM for ATP and 0.24 µM for AMP-PNP (Fig. 3C; Fig. S7). Importantly, we were unable to observe any polymerization of Salactin in the presence of ADP up to 10 µM Salactin protein (Fig. S8), indicating that ADP-bound Salactin has a much higher critical concentration than the non-hydrolyzed ATP-bound state (0.37 µM). Similar to what was observed with ParM and Alp7a (52, 58), the intermediate critical concentration of ~0.9 µM in the presence of hydrolyzable ATP likely reflects the “emergent critical concentration," the free monomer concentration that arises from the relative proportion of growing (ATP bound) and depolymerizing (ADP bound) filament ends. While the residual Salactin in the supernatant or the pelleting assays indicates that a fraction of the monomers are not polymerization capable, these experiments demonstrate that, under our buffer and salt conditions, ATP and ADP-bound Salactin monomers have at least a 30-fold relative difference in their affinity for filament ends, thereby giving the large energetic differential required for dynamic instability (52, 58).

We attempted to visualize Salactin filaments using negative stain electron microscopy, but the high salt in our buffer caused crystalline precipitates, inhibiting the observation of filaments, a common issue that arises when high salt is used with negative staining (59). We next attempted to visualize filaments *in vitro* using total internal reflection fluorescence (TIRF) microscopy. For this, we mixed 7 µM unlabeled Salactin with 0.34 µM cy3B-conjugated Salactin in the presence of 3 mM ATP. No filaments were observed under these initial conditions, but long Salactin bundles were observed in the presence of ATP but not ADP when we increased the macromolecular crowding with 17% polyethylene glycol (PEG) (Fig. 3D). These bundles did not display any depolymerization or dynamic instability during 1 hour of imaging, possibly caused by the crowding agents stabilizing filaments (60, 61) or from the fraction of labeled Salactin used (5%). This could also arise from the difference between the intercellular KCl concentration relative to the concentration in our buffers, as past work has shown that increasing salt changes the strength of hydrophobic and ionic interactions, which can affect interactions between monomers (62).

### Salactin influences the viability and DNA partitioning under low-phosphate growth conditions

We next conducted further phenotypic tests to gain insight into Salactin’s function. First, we saw no difference in the growth rates of ∆*salactin* and ∆*ura3* parent strains growing in rich media (hereafter called “standard phosphate” media) (Fig. S4A). Likewise, we saw no statistical difference in the motility between these strains (Fig. S9B).

**Fig 4:**
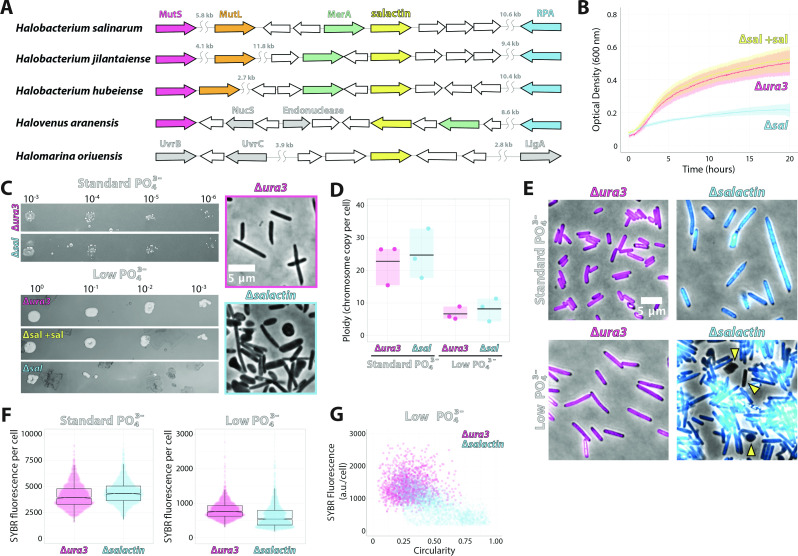
Cells lacking ∆*salactin* show defects in chromosomal partitioning and cell shape in low-phosphate media. Throughout, pink is ∆*ura3,* and blue is ∆*salactin*. (**A**) Synteny analysis of *salactin* (VNG_RS00630; VNG0153C) gene in closely related organisms to *H. salinarum*. (**B**) Growth curve showing the difference in the growth of ∆*salactin* cells in low-phosphate media taken from three technical replicates of three biological replicates*,* “*∆sal +* sal” indicates *∆salactin* cells complemented by exogenous expression of Salactin. (**C**) Spot dilutions of ∆*ura3* and ∆*salactin* cells grown on standard phosphate media and low-phosphate media indicating ∆*salactin* cells have a reduced viability in low-phosphate media relative to ∆*ura3* cells (left). Representative phase images of ∆*ura3* and ∆*salactin* cells from the spot dilution assay in low-phosphate media (right). Images are on the same scale, and the scale bar on the top right panel applies to the bottom right panel. (**D**) The bulk chromosomal number per cell by qPCR in standard phosphate and low phosphate reveals that there is no statistical difference (*P* > 0.05) in the average chromosomal number per cell regardless of media condition. Data were taken from three biological replicates. (**E**) Representative fluorescent images overlayed on the phase of ∆*ura3* and ∆*salactin* cells stained with SYBR-safe DNA stain in standard (left) and low-phosphate media (right). Yellow arrowheads indicate cells lacking chromosomal material. Images are on the same scale, and the scale bar on the first panel applies to all panels. (**F**) Quantification of the fluorescent intensity of SYBR-safe stained ∆*ura3* and ∆*salactin* cells in standard and low-phosphate media. (*P* < 0.0001 for both) Data were taken from three biological replicates. (**G**) SYBR fluorescence versus circularity of *ura3* and ∆*salactin* cells in low-phosphate media at stationary phase. Data were taken from three biological replicates.

Analysis of Salactin’s genomic context showed poor synteny conservation even within the Halobacterium genus (Fig. 4A), making it difficult to ascribe any function from its genetic neighborhood alone. However, in *H. salinarum* and its closest relatives, the *salactin* gene is in proximity to multiple genes involved in DNA replication and repair, suggesting that Salactin could be involved in a DNA-related process. As Haloarchaea are highly polyploid (63, 64), any phenotypes arising from a DNA-related process might not manifest unless ploidy is reduced. Past work has shown the ploidy of *Haloferax volcanii* drastically decreases (from 30 down to 2) when cells are grown in low-phosphate media, suggesting cells might limit and scavenge the excess chromosomal copies to increase their viability (65).

To test if ∆*salactin* cells showed a phenotype under decreased ploidy, we grew cells in a low-phosphate media. Because ∆*ura3* cells failed to grow in a medium lacking any phosphate, we grew our cells in a media that combined a defined media lacking phosphate (65) with 1% of rich standard phosphate (complete media, CM) media added to allow growth. In this growth condition, ∆*salactin* cells had a greatly decreased growth rate relative to ∆*ura3* cells, with ∆*salactin* cells considerably slowing down before one doubling (Fig. 4B). Spot dilution assays indicated that the decreased growth rate of ∆*salactin* cells in phosphate-limited media is likely due to a defect in viability: ∆*salactin* cells were indistinguishable from ∆*ura3* cells spotted on standard phosphate media but had at least two orders of reduced viability on low-phosphate plates. In addition, when ∆*salactin* and ∆*ura3* cells were imaged directly from colonies on low-phosphate spot dilution plates, there was a clear phenotypic difference in the shape of cells. ∆*ura3* cells were still consistently rod-shaped, while ∆*salactin* cells had cells that became rounder and amorphous (Fig. 4C). We confirmed that these phenotypes were linked to Salactin’s deletion, as we could rescue both viability and growth by complementing the ∆*salactin* strain with the expression of Salactin from a plasmid under the control of its native promoter (Fig. 4B and C).

To understand if the drop in viability in ∆*salactin* cells was caused by a difference in ploidy compared to the ∆*ura3* parental strain, we performed qPCR on cultures of each strain. In standard phosphate media, ∆*ura3* and ∆*salactin* populations showed ploidy levels indistinguishable from each other (23 ± 6.5 and 24 ± 7.4 chromosomal copies, respectively), consistent with what was previously described (66). In low-phosphate media, we observed the chromosomal copy number decrease approximately threefold for ∆*ura3* and ∆*salactin* cells (6.6 ± 2.0 and 8.2 ± 3.4 chromosomal copies, respectively) (Fig. 4D). Similar to the standard phosphate conditions, qPCR of ∆*ura3* and ∆*salactin* strains grown in low-phosphate media also did not show any statistically significant difference in ploidy, revealing that, at the population level, the number of chromosomes per OD remains constant in both growth conditions in the presence or absence of Salactin (Fig. 4D). This indicates that Salactin is likely not involved in DNA replication or the degradation of DNA that occurs in response to phosphate depletion.

Next, we examined how the absence of Salactin affected the distribution of DNA within single cells. We labeled the cellular DNA with SYBR-safe DNA stain and imaged cells with both phase contrast and widefield fluorescent microscopy. We performed these assays when cells were close to the stationary phase when cells had reduced ploidy (65, 66), as this might reveal greater phenotypic effects.

In standard phosphate media, while we could not observe a significant difference between the shape of ∆*ura3* and ∆*salactin* cells in the exponential phase (Fig. 1C), in the stationary phase, ∆*salactin* cells were significantly larger than ∆*ura3* cells (Fig. 4E; Fig. S10A). Also, ∆*salactin* cells accumulated slightly more (7.7%) SYBR fluorescence relative to ∆*ura3* cells (*P* < 0.0001) (Fig. 4F, left panel). In contrast, comparing the SYBR intensities between cells in standard phosphate and low-phosphate media revealed a clear downward shift for both ∆*ura3* and ∆*salactin* cells in low phosphate (Fig. 4F, right panel), in line with the reduced ploidy in our qPCR data when cells are shifted from standard to low-phosphate media (Fig. 4D).

In low-phosphate media, the amount of SYBR fluorescence in *∆salactin* cells was far more heterogeneous relative to ∆*ura3* cells: some cells appeared to have little to no chromosomal material, while others appeared to have greatly increased DNA staining (Fig. 4E; Fig. S10B). Quantifying the amount of SYBR fluorescence per cell revealed a significant difference (*P* < 0.0001) between ∆*salactin* and ∆*ura3* cells, where the ∆*salactin* strain displayed a more bimodal distribution, with a new second peak appearing at the low end of the distribution (Fig. 4F, right panel). ∆*salactin* cells also showed shape defects in low-phosphate media, with a large fraction of misshapen and amorphous cells, in contrast to the consistently rod-shaped ∆*ura3* cells (Fig. 4E). Interestingly, misshaped ∆*salactin* cells (higher circularity values) often correlated with reduced DNA staining (Fig. 4G). Given the average number of chromosomal copies per cell is the same between *ura3* and ∆*salactin* strains when assayed in bulk, these single-cell results suggest that Salactin might be required for correctly partitioning DNA between cells when ploidy is reduced, causing cells lacking Salactin to have less (or no) DNA, which might cause the cell shape defects and overall loss of viability. Importantly, Salactin-msfGFP filaments still showed dynamic instability when cells were grown in low-phosphate media (Fig. S11; Video S6).

## DISCUSSION

Our studies found that, in *H. salinarum,* Salactin shows dynamic instability, growing and shrinking out of the poles. This represents the third actin homolog shown to exhibit dynamic instability and the first characterization of the *in vivo* dynamics of any archaeal actin homolog. While not definitive, our data suggest that Salactin might be involved in partitioning DNA between daughter cells when chromosomes become limiting.

Similar to other dynamically unstable polymers (like microtubules, ParM, Alp7A, and PhuZ), Salactin filaments grow from one end while the incorporated monomers remain immobile within the growing filaments (49, 52, 58, 67, 68). Diffraction-limited Salactin filaments also appear to be composed of multiple filaments similar to what was observed for ParM filaments in *Escherichia coli* (53, 54). Likewise, as required for other dynamically unstable proteins, Salactin’s *in vitro* critical concentration in the non-hydrolyzed ATP state (~0.3 µM) is lower than the ADP form (>10 µM), providing the large energetic differential required for dynamic instability (52, 58). The critical concentration that arises in hydrolyzable ATP (~0.9 µM) likely reflects the relative proportions of growing and shrinking filaments in solution (52, 58). Given that different salt concentrations can substantially alter the critical concentration of eukaryotic actin (62), our *in vitro* measurements of Salactin’s critical concentration conducted at much lower salt concentrations are likely not the same as those inside the cell and might explain why we were unable to recapitulate dynamic instability *in vitro*. However, even at our reduced *in vitro* salt concentrations, these measures suggest that there exists a large difference in the ADP and non-hydrolyzable ATP critical concentrations as required for dynamic instability in the cell.

Cells lacking Salactin displayed substantial phenotypes only when cells were grown in low-phosphate media. *H. volcanii* has been shown to reduce its number of chromosomes from 30 down to as low as 2 in low-phosphate media (65). Similarly, we also observe a reduction in *H. salinarum’s* ploidy and amount of DNA from 23 to 25 chromosomes down to 7–8 chromosomes in low-phosphate media. Given ∆*salactin* cells show a DNA partitioning defect in low phosphate, it is likely that Salactin could be part of a DNA segregation system that, similar to low-copy plasmids, is required when the number of DNA chromosomes is not sufficiently high to be passed to both daughter cells by random chance. An inability to partition limiting chromosomes could create the apparent anucleate ∆*salactin* cells, which could possibly explain their defects in bulk growth and viability, as well as the apparent correlation between cells lacking DNA signal having a perturbed cell shape. Alternatively, given Salactin’s chromosomal proximity to multiple DNA repair enzymes in *H. salinarum* and its closest relatives (Fig. 4A), Salactin could also be involved in DNA repair and the SOS response.

Given all other known dynamically unstable filaments [microtubules (69), ParM (52), Alp7A (11), and PhuZ (67)] are involved in DNA partitioning, our results suggest that Salactin filaments could partition archaeal DNA by their growth or catastrophe, pulling from some “kinetochore-like” region on the archaeal chromosome. Because we currently lack the tools to label discrete loci on chromosomes, we were unable to verify our model, but future studies that simultaneously image Salactin filaments and discrete chromosomal loci could test this model.

## MATERIALS AND METHODS

### Strains, plasmids, and primers

*Halobacterium salinarum* NRC-1 (ATCC 700922) was the wild-type strain used in this study. Tables S4, S5, and S6 list strains, plasmids, and primers used in this study. For more detailed plasmid construction, see Methods in supplemental material. The pRrpa plasmids were created from a modified version of the pMTFChis (70), where the original promoter, P_fdx_, is replaced with another promoter, P_rpa_. Plasmid constructs for the transformation of *H. salinarum* were generated by isothermal Gibson assembly (71) of: (i) the PCR fragments (amplified by KAPA Biosystems DNA polymerase [VWR] and gel extracted) and (ii) the linear plasmid. Plasmids were propagated in *E. coli* DH5. Proteins were tagged with fluorescent proteins as C-terminal fusions using a 15 amino acid linker (LEGSGQGPGSGQGSG). Plasmids were verified by Sanger sequencing of the locus in the plasmid. Plasmids were transformed into *H. salinarum* using a polyethylene glycol 600 spheroplast protocol (72, 73) and selected using mevinolin. The protocol used for protein overexpression and purification using the his6SUMO construct (pSUMO) (74) was as described by Stoddard and colleagues (75).

### Media and growth conditions

*H. salinarum* strains were routinely grown, unless otherwise specified, using a nutrient-rich medium, CM (complete media) medium (250 g/liter NaCl [Fisher Scientific]; 20 g/liter MgSO_4_·7H_2_O [Fisher Scientific]; 3 g/liter trisodium citrate [Fisher Scientific]; 2 g/liter KCl [Fisher Scientific]; 10 g/liter bacteriological peptone [Oxoid]; pH 6.8). Media were supplemented with 50 g/mL uracil (Sigma) to complement the uracil auxotrophy of the *Δura3* background. All growth was performed at 42°C in a roller drum. Self-replicating *H. salinarum* plasmids were maintained using 1 g/mL mevinolin in liquid culture. Cells were grown at 37°C during live-cell microscopy. *E. coli* was grown in an Luria-Bertani (LB) medium with carbenicillin (50 g/mL; Sigma) to maintain plasmids. For *H. salinarum* growth in low phosphate, a phosphate-free media, which was adapted from Zerulla and colleagues, was prepared (65) (10 mM NH_4_Cl; 0.5% glucose; 25 mM CaCl_2_; 10 mM trisodium citrate·2H_2_O; 25 mM KCl; 100 mM MgSO_4_·7H_2_O; 4.2 M NaCl; trace metal solution; vitamin solution; pH 6.8). Trace metal and vitamin solutions were prepared as described by de Silva and colleagues (76). The low-phosphate condition consisted of the phosphate-free medium mixed with rich CM 99:1 prior to *H. salinarum* culturing.

### Data visualization

Data were plotted with GraphPad Prism (Prism 9 for macOS version 9.3.1, PlotsOfData [https://huygens.science.uva.nl/PlotsOfData/] [77] and PlotTwist [https://huygens.science.uva.nl/PlotTwist] [78]).

### Genomic neighborhood analysis

Synteny of *salactin* across different haloarchaeal genomes was performed using Syntax (https://archaea.i2bc.paris-saclay.fr/SyntTax/) (79). Salactin protein sequence from *H. salinarum* was used as bait in search against deposited haloarchaeal genomes with a 20% minimal normalized genomic BLAST score.

### Whole-genome sequencing

*H. salinarum* was grown to the mid/late-logarithmic phase (optical density at 600 nm [OD_600_ ], ~0.7), and 1.5 mL was pelleted by centrifugation and stored at −20°C until processed. Cells were lysed in ddH_2_O, DNA was extracted using a phenol-chloroform method in phase lock gel tubes, and ethanol precipitated and washed. The DNA pellet was resuspended in Tris EDTA (TE) buffer and quantified using a NanoDrop. Samples were submitted to the Bauer Core Facility in the FAS Division of Science at Harvard University for Nextera XT 1/4 volume queued library prep and an Illumina MiSeq v2 run for whole-genome sequencing. Sequences were trimmed by BBDuk trimmer prior to whole-genome assembly using Geneious *de novo* assembly of the paired-end plugin. The results of this analysis can be found in File S3.

### Phylogenetic analysis

We performed iterative HMM searching to retrieve *salactin* homologs from a collection of 700 representatively sampled archaeal and bacterial proteomes (80). Beginning with the *salactin* sequence record (WP_010902067.1) from *H. salinarum*, we retrieved all HMMsearch r hits (HMMer 3.3.2) (81) with an *e*-value < 1*e*-7. These sequences were aligned using MAFFT (L-INS-i) (82) and then used to construct a new HMM profile in order to retrieve additional homologs from the proteomes, and three additional representative sequences from methanotectans other than Haloarchaea were identified using NCBI BLASTp and included (RUM33890, PXF51824.1, and OYT66940.1). We also performed additional HMM searches and found sequences for other potential homologous gene families (see the supplemental material): Actin, Crenactin, Lokiactin, MreB, FtsA, DnaK/Hsp70, ParM, and MamK. After removing any duplicate identical sequences and adding the initial annotated sequences used to construct the HMM profiles to aid with annotation, these 1,838 sequences were then aligned using MAFFT (auto) (82), and an expanded tree was inferred using IQTREE2 (83) LG + G, 10,000 ultrafast bootstraps (39) (Fig. S1B). Based on these results, we then inferred a more focused tree including crenactin, Salactin*,* and MamK sequences, with the actin/lokiactin sequences included for an outgroup (Fig. S1C), then a tree focusing only on *salactin* and MamK (Fig. S1D), and then finally with only the Salactin sequences (Fig. S1E). These trees were inferred using the best-fitting model under BIC in IQ-TREE2 (82). LG + C60 + G was chosen for the *salactin*-only tree, whereas LG + C20 + F + G was selected for the other two trees; we used 10,000 ultrafast bootstrap replicates (39) each time. All trees, alignments, HMM profiles, HMM output tables, and retrieved sequences are available in File S1.

### Growth curves

1. Rich media: liquid cultures were grown in CM (3 mL) from single colonies until saturation. Cells were then diluted to an OD_600_ of 0.025, representing time 0 for all growth curves. OD measurements were taken by hand every 6–12 hours throughout the growth curve.
2. Low-phosphate media: cultures were grown in a rich medium (CM) as described above, then centrifuged at 4,000 *× g* for 5 minutes and washed three times with phosphate-free media. After washing, cells were resuspended to an initial OD_600_ of 0.2 in low-phosphate media. Two hundred microliters of each sample (and fresh media as a blank) were then transferred to a 96-well plate to be read in a BioTek EPOCH2 plate reader. Growth curves were obtained by taking OD_600_ readings every 30 minutes for 24 hours under continuous orbital shaking (425 cpm) at 42°C. Data were plotted using PlotTwist (https://huygens.science.uva.nl/PlotTwist) (78).

### Motility assay and diameter measurements

Liquid cultures were grown in CM from single colonies until saturation. Cells were then diluted to an OD_600_ of 0.025 in CM and grown to an early log phase (OD_600_ 0.1–0.3). The OD_600_ of these cells was normalized, and then the cells were stabbed onto a 0.3% 1:10 CM (same composition as CM but 1/10 Oxoid peptone) agar plate for the motility assay. The diameter was measured by hand on day 6 of incubation at 42°C in a thick plastic bag to control the loss of humidity.

### Spot dilution viability assay

∆*ura3* and ∆*salactin* cells were grown in rich media (CM) until the exponential phase (OD_600_ = 0.5) and then serially diluted in fresh rich media. Two microliters of each diluted culture were spotted onto rich CM and low-phosphate agar plates and incubated at 42°C.

### Ploidy determination by qPCR

Standard curves were generated with isolated gDNA from cultures grown in the spot dilution assay described above. Genomic DNA was isolated using the Quick-DNA Fungal/Bacterial Miniprep Kit (Zymo Research). Concentrations were determined by NanoDrop (Thermo Scientific 2000), and each sample was diluted to 0.4 ng/µL. Samples were serially diluted so their final working concentrations fell within the range of the generated standard curve. The standard curve was created using a 270 bp PCR product (66). oJM220 and oJM221 primers were designed using NCBI’s PCR primer design tool (https://www.ncbi.nlm.nih.gov/tools/primer-blast/). A standard PCR reaction was performed at 95°C for 2 minutes (denature), followed by 35 cycles of 15 seconds at 95°C (denature), 15 seconds at 55°C (annealing), and 20 seconds at 68°C (extension). The 270 bp amplicon was purified from an agarose gel using the Zymoclean Gel DNA Recovery Kit (Zymo Research). qPCR was performed on a 10-fold dilution series of the purified product from 0.1 ng to 1 × 10^−6^ ng of template. Template concentration versus Cq values were plotted, and a linear line was fit to the data. The equation of this line was used to determine the DNA amounts in the ploidy experiments. The qPCR reactions were performed using a clear, 96-well PCR plate (Olympus Plastics). To each well, 7.5 µL of a master mix was added, consisting of SYBR Green/dNTPs/Taq polymerase/and primers at 0.25 µM. A melt curve was then determined by heating the samples from 65°C to 95°C in 0.5°C steps. The qPCR was performed in the CFX96 Real-Time System (Bio-Rad). Data analysis was performed, and Cq values were determined using the CFX Maestro software (Bio-Rad). DNA was extracted from a total of three biological replicates, and all samples were run in triplicate with water acting as a template control. Calculated Cq values were compared to a standard curve and divided by the colony-forming unit from initial cultures to determine the copy number of the main chromosome per cell in each sample.

### RNA extraction and sequencing

Strains hsJZ52 (three colonies) and ura3 were grown in HCM to an OD_600_ of 0.6. RNA extraction was performed as described in reference 84 with three 75% ethanol washes. Ribodepletion was done using *Halobacterium salinarum*-specific probes (see below). Purified RNA was sent to SeqCenter for ribosome depletion and sequencing. Results were mapped to the NRC-1 genome and analyzed using Geneious 2002.2. Transcripts per million, SeqCenter quality control files, and raw sequencing data (.fastq files and quality control files), as well as the ribodepletion probes, can be found in this folder. The complete raw RNA-seq data sets can be found in NCBI GEO online repositories: PRJNA986495, PRJNA986501, PRJNA986500, and PRJNA986487.

### Imaging

Unless otherwise noted, strains were grown in liquid culture (3 mL CM) from single colonies until saturation. Cells were then diluted to an OD_600_ of 0.1 and grown to exponential phase (OD_600_ 0.5–0.8) before the start of all imaging experiments.

1. Cell size and shape measurements. Strains: ∆*ura3,* ∆*salactin*. For imaging, 5 µL culture aliquots were immobilized on no. 1.5 cover glass under a CM agar pad (0.3%, wt/vol). Phase-contrast images (100-ms exposure) were collected on a Nikon TI microscope equipped with a 6.45-µm-pixel Andor Clara camera and a Nikon × 100 numerical aperture (NA) 1.4 objective. The phase-contrast images were segmented manually, and size and shape measurements were obtained using Fiji’s measure function. The difference between the two groups was analyzed with unpaired *t*-tests with Welch’s correction using Prism 9 for macOS version 9.3.1 for all statistical analyses unless otherwise stated.
2. Imaging of dynamics. Strains: WT + *prpa-salactin-msfGFP* and *prpa-salactin-halotag*, ∆*salactin + prpa-salactin-msfGFP*, and *prpa-salactin-halotag*. For imaging, 5 µL culture aliquots were immobilized on no. 1.5 cover glass under a CM agar pad (0.3%, wt/vol). Cells were imaged in a Nikon Eclipse Ti microscope with a 6.5-m pixel ORCA-Flash4.0 V2 sCMOS Hamamatsu camera and a Nikon 60 NA 1.4 phase-contrast objective for comparing dynamics between conditions and a 100 NA 1.45 phase-contrast objective for dynamic phenotypes. Fluorescence excitation was achieved using an MLC4008 laser launch (Agilent), with a 488 nm laser used for msfGFP imaging and a 561 nm laser used for imaging of JF549 conjugated to the HaloTag. Using fluorescence highly inclined and laminated optical sheet (HiLo) microscopy, images were captured every 30 seconds for 30 minutes to 1 hour at 40% laser power. Exposure times for fluorescence were 100 and 200 ms for the 488 and 561 nm laser, respectively. The polymerization rates, length, and time for catastrophe were measured by kymograph analysis. Kymographs were created from time lapses of fluorescently labeled Salactin filaments by manually drawing regions of interest (ROIs) along the long axis of the cells in Fiji or along the filament (whichever created clearer kymographs). Regions of these kymographs containing right triangles represented dynamic instability and were measured manually in Fiji for the dynamic measurements. For the first exponential distribution analysis, the time for catastrophe is plotted as a relative frequency histogram with a bin size of 150 and fitted to a one-phase decay exponential in the “non-linear regression (curve fit) analysis” in Prism 9 (https://www.graphpad.com/). For the second fitting method, the logarithm of frequency is used to create a linear fit using the “simple linear regression analysis” in Prism 9 (https://www.graphpad.com/).
3. Single-molecule imaging. Strains: WT + *prpa-salactin-halotag*. To decrease autofluorescence, liquid cultures were grown in HS-Ca media, which was modified from the Hv-Ca (85) (3 mL) (25% BSW [240 g/L NaCl, ChemSupply: SA046], 30 g/L MgCl_2_·6H_2_O [Sigma: M2393]), 35 g/L MgSO_4_·7H_2_O [Sigma: V800245], 7 g/L KCl [Sigma: V800245], 20 mL 1 M Tris-HCl pH7.4 [ChemSupply: TA034]), 0.5% (wt/vol) Casamino acids (5 g/L [Oxoid: LP0041]), from single colonies until saturation. Cells were then diluted to an OD_600_ of 0.1 to grow to late exponential (OD_600_ 0.75–0.85). Cells were conjugated with a mixture of JF dyes (86) to perform single-molecule and whole-cell labeling to verify the localization of the single molecules. JF dyes were added to the growth media 15 minutes before imaging. The ratio of the dyes was 1:20 JF549:JF505; 1.25 nM of JF549 and 25 nM of JF505 were used. For imaging, 5 µL culture aliquots were immobilized in Matek dishes under an HS-Ca agar pad (0.3%, wt/vol) to optimize signal-to-noise. Cells were imaged in a Nikon Eclipse Ti microscope with a 6.5-m pixel ORCA-Flash4.0 V2 sCMOS Hamamatsu camera and a Nikon 100× NA 1.45 phase-contrast objective. Fluorescent images were obtained using a MLC4008 laser launch (Agilent), with a 488 nm laser for JF505 imaging and a 561 nm laser for the JF549 imaging. Using HiLo microscopy, fluorescent images were captured every 5–10 seconds for 30 minutes to 1 hour. Exposure times for fluorescence were 1 s (561 nm laser) and 250 ms (488 nm laser).
4. DNA labeling in live cells. Cells were grown in rich media until the exponential phase (OD_600_ ~ 0.5), then centrifuged at 4,000 *g* for 5 minutes and washed three times with phosphate-free media. After washing, cells were resuspended to an initial OD_600_ of 0.2 in rich or low-phosphate media and grown to saturation. Live-cell DNA labeling was done by adding SYBR Safe DNA Gel Stain (Invitrogen) to a final 10^6^-fold dilution. Cells were then incubated for 5 minutes at room temperature and promptly imaged. The difference between the DNA labeling of the two groups was analyzed using the Mann-Whitney test using Prism 9 for macOS version 9.3.1.
  Visualization of filaments in TIRF. Seven micromolar of Salactin was mixed with 1/20th of stained Salactin-GSKCK. To visualize Salactin fluorescently, Salactin-GSKCK was conjugated with cy3B mono maleimide. To conjugate the protein to the dye, the protein was pre-reduced with 5 mM Tris(2-carboxyethyl)phosphine hydrochloride (TCEP) for 30 minutes. TCEP was removed with a desalting column (NAP-5), and 5× excess dye was added and incubated for 10 minutes on ice. The reaction was quenched by adding 10 mM DL-dithiothreitol (DTT). The protein was hard spun on a TLA100 rotor at 90,000 rpm for 20 minutes. The supernatant was loaded on a NAP-5 desalting column, and concentration was measured based on the A_280_. Polymerization reactions were initiated upon adding 3 mM MgATP, or MgAMPPNP, with 17.6% PEG8000 to protein and incubated at 37°C for 3 hours. Without any PEG, filaments could not be visualized under TIRF. For imaging, 5 µL of protein solution was immobilized between two no. 1.5 cover glasses (22 × 60 mm base and 18 × 18 mm cover) and visualized using TIRF microscopy on a Nikon Eclipse Ti microscope with a 6.5-m pixel ORCA-Flash4.0 V2 sCMOS Hamamatsu camera and a Nikon 100 NA 1.45 phase-contrast objective. Fluorescent images were obtained using an MLC4008 laser launch (Agilent) with a 561 nm laser to image the cy3B dye. Images were captured with a 200 ms exposure time at 30% laser power.

### Protein purification

BL21 (DE3) Rosetta containing the his6SUMO fusion plasmid was grown to OD_600_ ~ 0.6 and induced for 4 hours with 0.4 mM IPTG at 37°C. Cell pellets were resuspended in I0 buffer (50 mM Tris, 300 mM KCl, 1 mM MgCl_2_, 10% glycerol. Added before use: 0.5 mM TCEP and 0.2 mM ATP) and stored at −80°C until use. Cells were lysed using a Misonix Sonicator. His6SUMO fusion products were then purified using a 5 mL HisTrap HP (GE Healthcare) on an AKTA pure with stepwise imidazole·HCl increases from 15, 35, 50, 65, to 80 mM and a final gradient to 500 mM imidazole·HCl (I500). The His6SUMO tag was cleaved off using the Ulp1 protease (74) during dialysis of the protein back into I0. Using gravity, proteins were then run through a 4 mL bed of HisPur Ni-NTA resin (Thermo Scientific) with a wash step of I0 buffer (where the desired cleaved protein will come out) and an I500 buffer elution step. Proteins were further purified using a 5 mL HiTrap Q FF (GE Healthcare) on an AKTA pure. Protein was dialyzed into starting buffer for anion exchange (20 mM Tris pH 8, 30 mM KCl, and 1 mM MgCl_2_. Added before use: 0.5 mM TCEP and 0.2 mM ATP) and bound to the HiTrap Q. The column was washed with starting buffer (5CV), 20% of elution buffer (5CV) (20 mM Tris pH 8, 1 M KCl, and 1 mM MgCl2. Added before use: 0.5 mM TCEP and 0.2 mM ATP), eluted using a gradient from 20% elution buffer to 65% elution buffer, and washed with 100% elution buffer. Fractions with protein were pooled and concentrated using a 10k MWCO PES Pierce Protein Concentrator (Thermo Scientific). Proteins were buffer exchanged using a prepacked PD-10 desalting column into HP buffer (2.9 M KCl, 5 mM MgCl2, and 10 mM HEPES, pH 7, and 0.2 mM EGTA. Added before use: 0.2 mM ATP and 0.5 mM TCEP). The protein concentration was determined using a Pierce BCA Protein Assay kit (Thermo Scientific) due to the low A_280_ signal from the absence of tryptophan in the protein. Twenty-five percentage of glycerol was added to the protein, and aliquots were snap-frozen in liquid nitrogen and stored at −80°C until needed.

### Malachite green assay

Malachite green assays were performed using a malachite green phosphate assay kit (MAK307-1KT: Sigma-Aldrich). Polymerization reactions were started by mixing at least 5.5 µM protein (unless otherwise stated) with 0.2 mM ATP and heated to 37°C for varying times. The reaction was stopped by adding 25 µM EDTA and 75 µM sulfuric acid. The reaction was spun down for 5 minutes at 16 × *g*, the supernatant was taken, and phosphate was measured using the malachite green assay kit. The phosphate release values were calculated by subtracting from the value at each time point of a “blank” reaction containing only nucleotide (no protein) from the values from the reaction containing both protein and nucleotide at the same time point. Resultant values less than zero were assigned as zero.

### Pelleting assay

Unless otherwise noted, polymerization reactions were done in HP buffer with 2.9 M KCl and contained 2.5 mM MgATP or MgAMPPNP. When testing other nucleotides, 2.5 mM of the nucleotide is used. Protein was exchanged into HP buffer without ATP and initiated upon adding MgATP or MgAMPPNP. Polymerization reactions were run for 3 hours at 37°C . Polymerization reactions were spun in a TLA100 (Beckman) at 436,000 × *g* for 30 minutes at 37°C. Supernatants were removed and added to an equal volume of 2× SDS buffer. Pellets were resuspended by heating at 65°C in two volumes of 1× SDS buffer. Fractions were subjected to SDS-PAGE and stained with SYPRO Orange (ThermoFisher). The gel was visualized on a c200 Azure gel imaging station, with the EPI Blue LED, with a 470 nm wavelength, and band intensities were quantified in ImageJ (87). Note that more qualitative data, like the pelleting comparison for different nucleotides, were taken on a blue box using an iPhone camera.

### Fluorimeter experiments

Protein was exchanged into HP buffer without ATP and initiated upon adding MgATP or MgAMPPNP. Light scattering polymerization reactions were initiated by mixing protein with 17.6% PEG8000 and 3 mM MgATP or 2 mM MgAMPPNP and incubated at 37°C for 3 hours. Without any PEG, there would be no signal of polymers forming after 3 hours of incubation, even though pelleting indicates that polymerization occurs. All light scattering experiments were endpoint assays at 3 hours. The 90° scattering of the solution at 315 nm was measured using a Fluorolog-3 (Horiba).

## Data Availability

The data sets generated and/or analyzed during the current study are available from the corresponding author upon reasonable request. Supplemental files are available in the Dataverse repository at https://doi.org/10.7910/DVN/JPN2C4.

## SUPPLEMENTAL MATERIAL

Supplemental material mbio.02272-23-s0001.pdf

Video S1 mbio.02272-23-s0002.mov Salactin-msfGFP and expressed on top of the native copy, visualized by near-TIRF fluorescent microscopy.

Video S2 mbio.02272-23-s0003.avi Salactin-HaloTag and expressed on top of the native copy, visualized by near-TIRF fluorescent microscopy.

Video S3 mbio.02272-23-s0004.avi Salactin-HaloTag labeled with JF549 that was expressed on top of the native copy.

Video S4 mbio.02272-23-s0005.avi Salactin-HaloTag expressed as a sole copy visualized by near-TIRF fluorescent microscopy.

Video S5 mbio.02272-23-s0006.avi Salactin-msfGFP expressed as a sole copy visualized by near-TIRF fluorescent microscopy.

Video S6 mbio.02272-23-s0007.avi Salactin-msfGFP expressed on top of the native copy (hsJZ52).

ASM does not own the copyrights to Supplemental Material that may be linked to, or accessed through, an article. The authors have granted ASM a non-exclusive, world-wide license to publish the Supplemental Material files. Please contact the corresponding author directly for reuse.
