# Supplementary material for: Salactin, a dynamically unstable actin homolog in Haloarchaea
Source: mBio. 2023 Nov 15;14(6):e02272-23. doi: 10.1128/mbio.02272-23 (PMC10746226; doi:10.1128/mbio.02272-23)
Supplement: Supplemental material [file mbio.02272-23-s0001.pdf]

SUPPLEMENTAL FIGURES

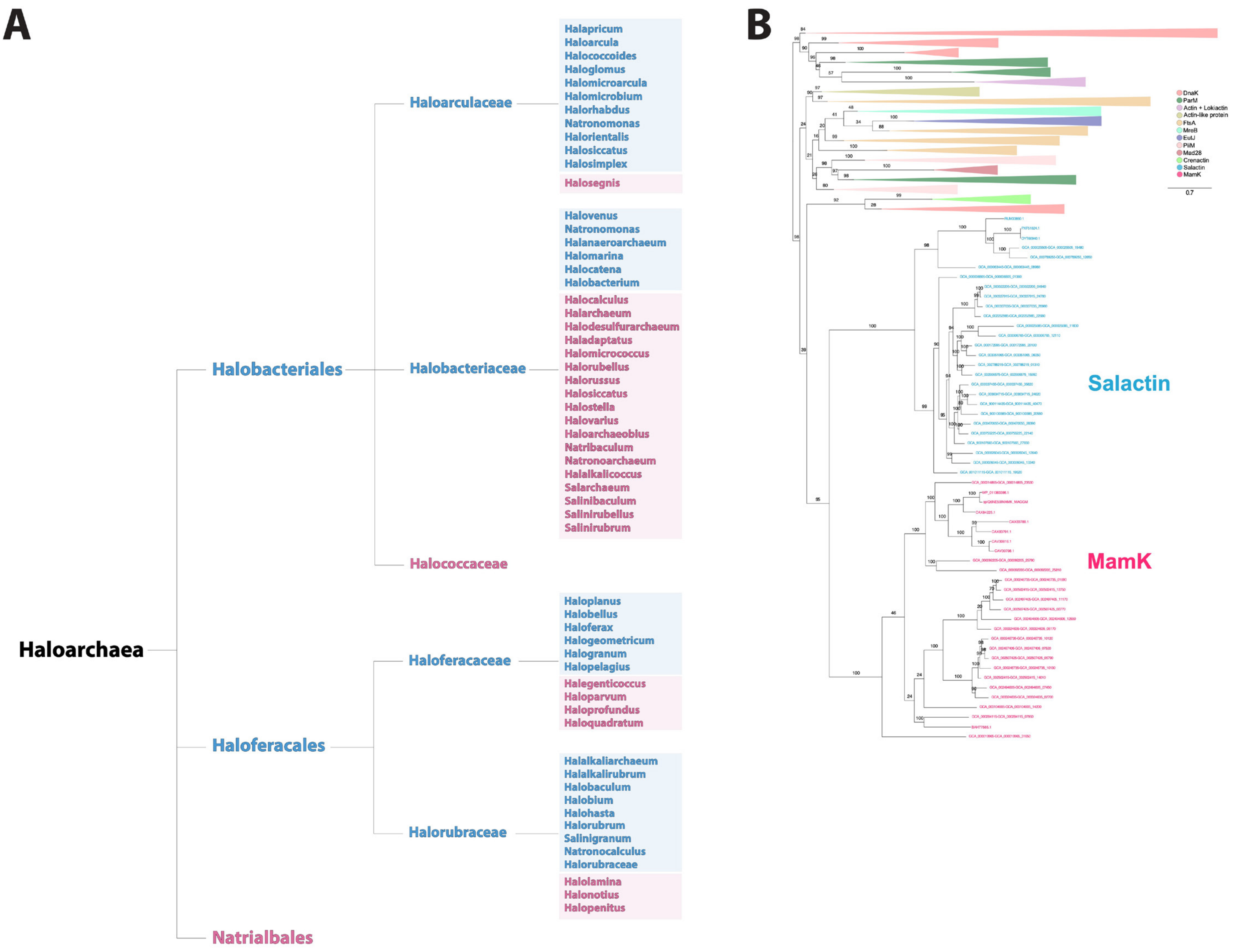

Supplemental Figure S1. Distribution of salactin across Haloarchaea and relative to other actin-like proteins. All alignments and trees are available in supplemental file S1. **(A)** Non-phylogenetic organogram representing the presence (cyan) and absence (pink) of salactin supported by JackHMMER. **(B)** Phylogeny of actin-fold proteins, focusing on salactin. Clades are colored according to their annotations: DnaK, ParM, Actin, Actin-like proteins, FtsA, MreB, EutJ, PiiM, Mad28, Crenactin, Salactin, Mamk. Aside from salactin and MamK these are collapsed for visualization purposes. The tree is unrooted but has been drawn as though rooted on the branch, separating the vast majority of DnaK sequences to aid visualization. Branch lengths are proportional to the expected number of substitutions per site, as indicated by the scale bar. The tree was inferred under the LG+G model in IQ-TREE2(1). Support values are calculated from 10,000 ultrafast bootstrap replicates in IQ-TREE2 (2).

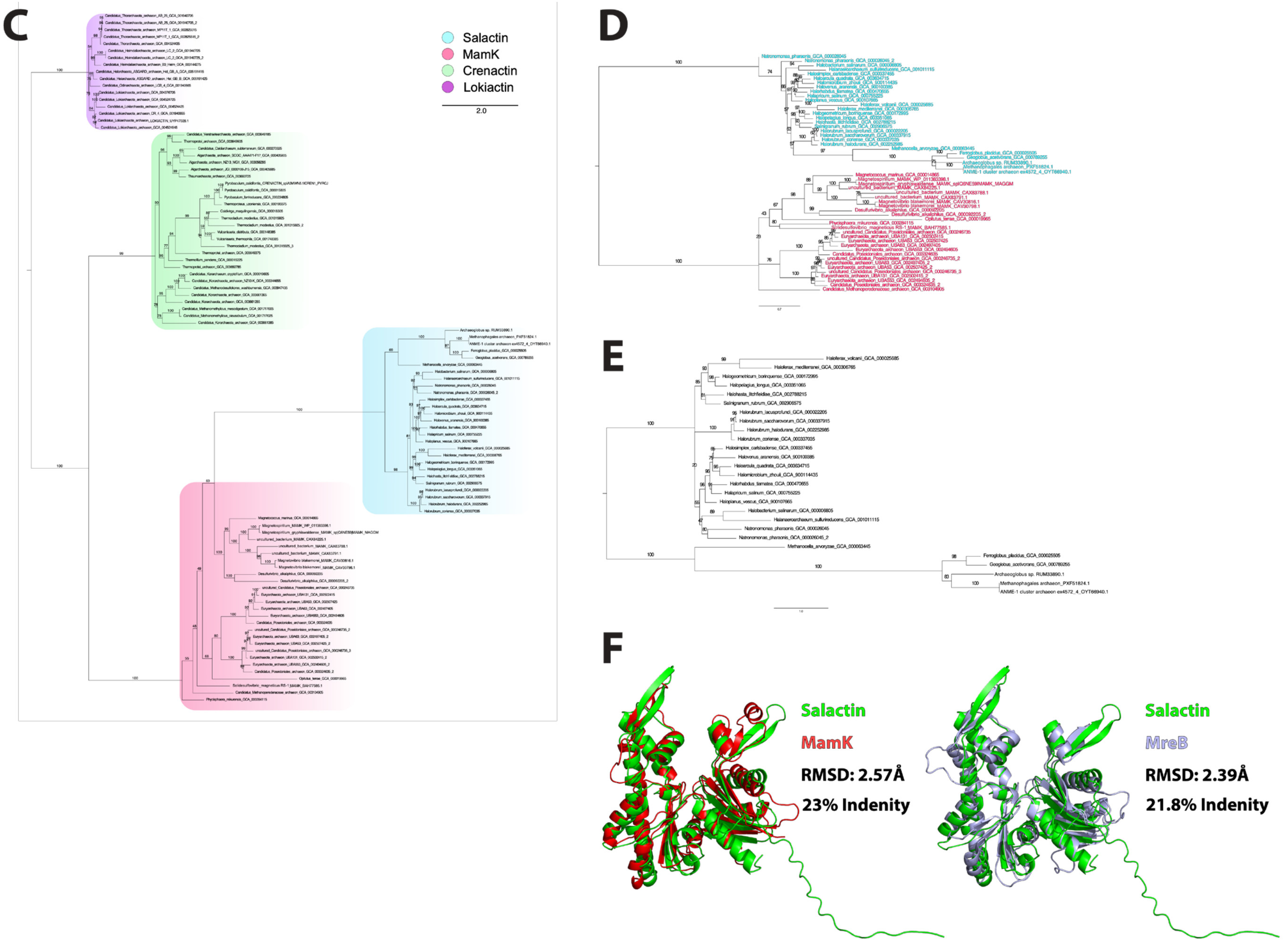

Supplemental Figure S1C-E. (C) Phylogeny of salactin and the most closely related actin subfamilies MamK, and Crenactin, with Asgard archaeal actins as a potential outgroup. The tree is unrooted. Branch lengths are proportional to the expected number of substitutions per site, as indicated by the scale bar. The tree was inferred under the LG+C20+F+G model in IQ-TREE2 (1), which was the best-fitting model according to the BIC criterion. Support values are calculated from 10,000 ultrafast bootstrap replicates (2) in IQ-TREE2. **(D)** Phylogeny of salactin (cyan) and MamK (red). The tree is unrooted. Branch lengths are proportional to the expected number of substitutions per site, as indicated by the scale bar. The tree was inferred under the LG+C20+F+G model in IQ-TREE2 (1), which was the best-fitting model according to the BIC criterion. Support values are calculated from 10,000 ultrafast bootstrap replicates (2) in IQ-TREE2. **(E)** Phylogeny of salactin homologs in Methanotecta. The tree is unrooted. Branch lengths are proportional to the expected number of substitutions per site, as indicated by the scale bar. The tree was inferred under the LG+C60+G model in IQ-TREE2 (1), which was the best-fitting model according to the BIC criterion. Support values are calculated from 10,000 ultrafast bootstrap replicates (2) in IQ-TREE2. **(F)** Backbone alignments of the predicted Salactin structure by Alphafold (ID: Q9HSN1) with (*left*) MamK (from *Magnetospirillum magneticum* PDB ID: 5LJV) and *(right)* MreB (from *Caulobacter crescentus* PDB ID: 4CZL).

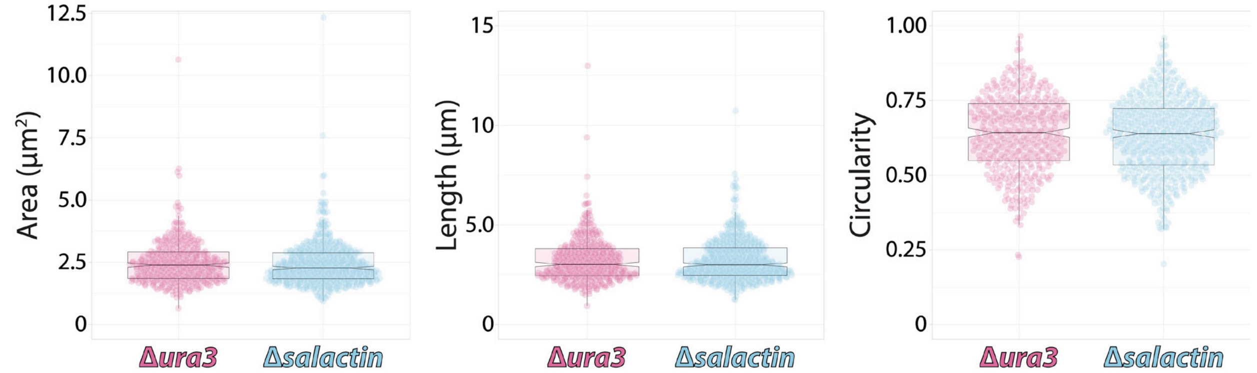

Supplemental Figure S2. Violin plots showing that Δ*salactin* and Δ*ura3* cells do not show any statistical difference in their (*left*) area (p-value = 0.2721), (*mid*) length (p-value = 0.9198), or (*right*) circularity (p-value = 0.3924). Data was taken across 3 biological replicates with a total N of 375 and 493 for Δ*ura3 and* Δ*salactin* cells, respectively.

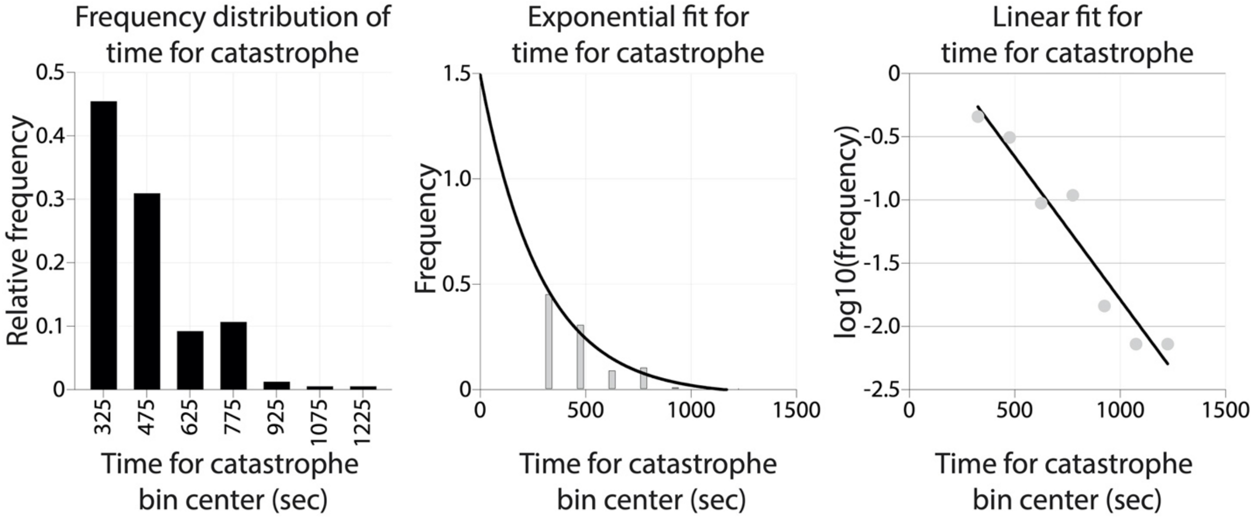

Supplemental Figure S3. Catastrophe time distribution analysis. Time for catastrophe plotted as a relative frequency bar graph with 150 bin sizes (*left*) and fitted to a one-phase decay exponential (*middle*). R-squared value of linear fit (*left*) = 0.9335. N = 134.

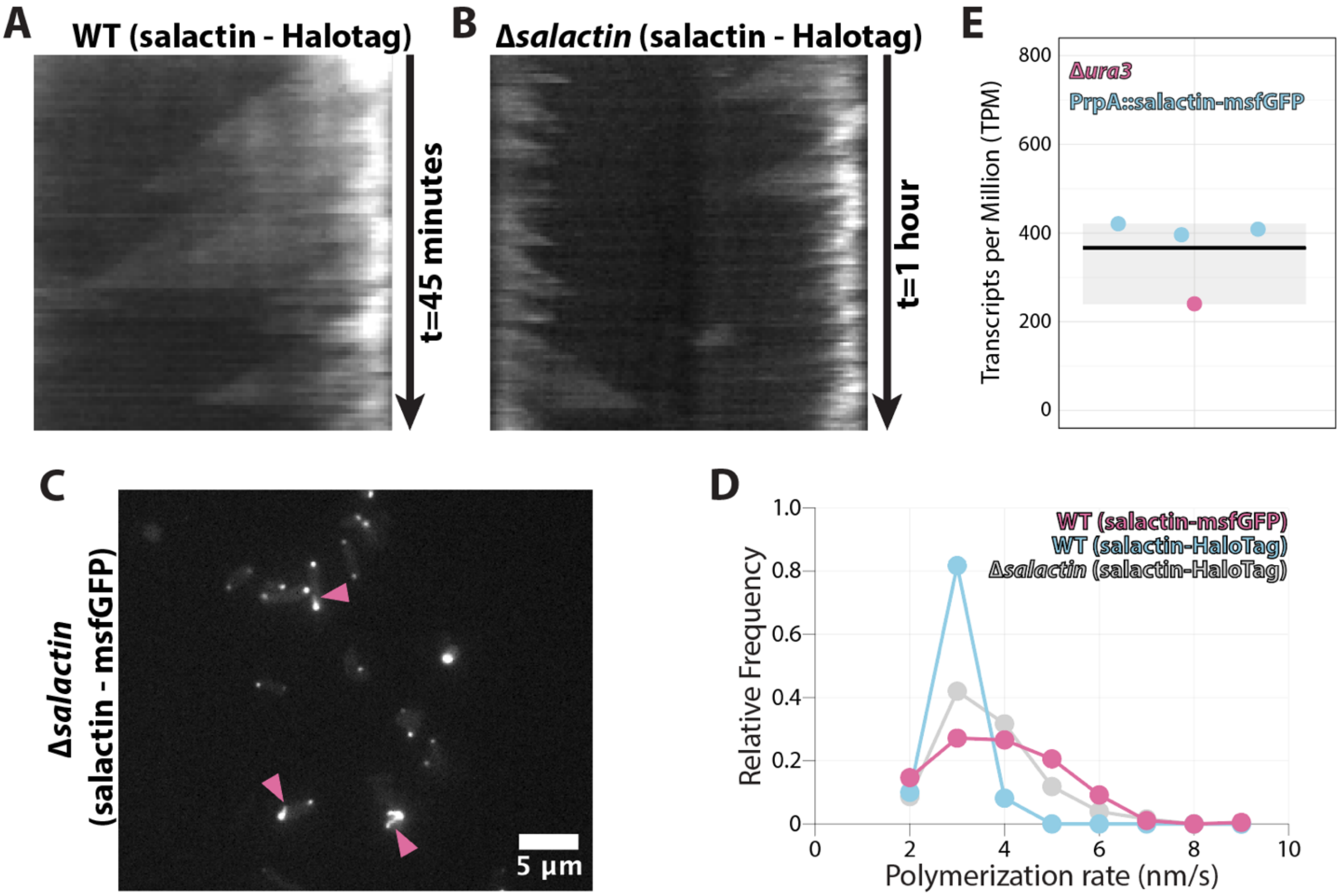

Supplemental Figure S4. Dynamics of Salactin fused to different tags. (A) Kymograph of Salactin-HaloTag (strain hsJZ86) also expressing the native *salactin* copy. (B) Kymograph of Salactin-HaloTag expressed in Δ*salactin* cells (strain hsJZ106). (C) Fluorescent images of Salactin-msfGFP expressed in Δ*salactin* cells (strain hsJZ95). The pink arrow points toward the appearance of filaments. (D) Histogram of the relative frequencies for polymerization rates comparing wild type + Salactin-msfGFP (strain hsJZ52), wild type + Salactin-Halotag (strain hsJZ86), Δ*salactin* + Salactin-HaloTag (strain hsJZ106). The wild type + Salactin-HaloTag has a significantly (p-value <0.0001) slower polymerization rate, while the other two have comparable polymerization rates. (E) RNA-seq from total mRNA extracted from strains ura3 and hsJZ52. Salactin transcriptional measurements showing the overexpression levels were calculated by dividing the number of sequenced salactin reads by the total reads from each dataset. N = 184 for WT (salactin-msfGFP), 110 for WT (Salactin-Halotag), and 126 for Δ*salactin* (Salactin-Halotag).

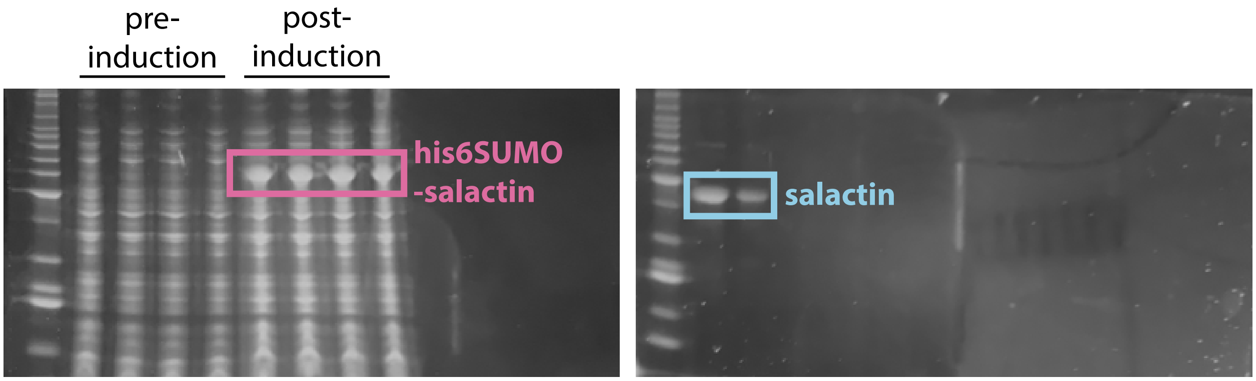

Supplemental Figure S5. SDS-PAGE gels of the purification protocol at the beginning (induction) and end (purified protein). Pre- and post-induction of Salactin expressed in *Escherichia coli* (*left*). Final purified protein used for *in vitro* assays (*right*). All gels are stained with SYPRO Orange.

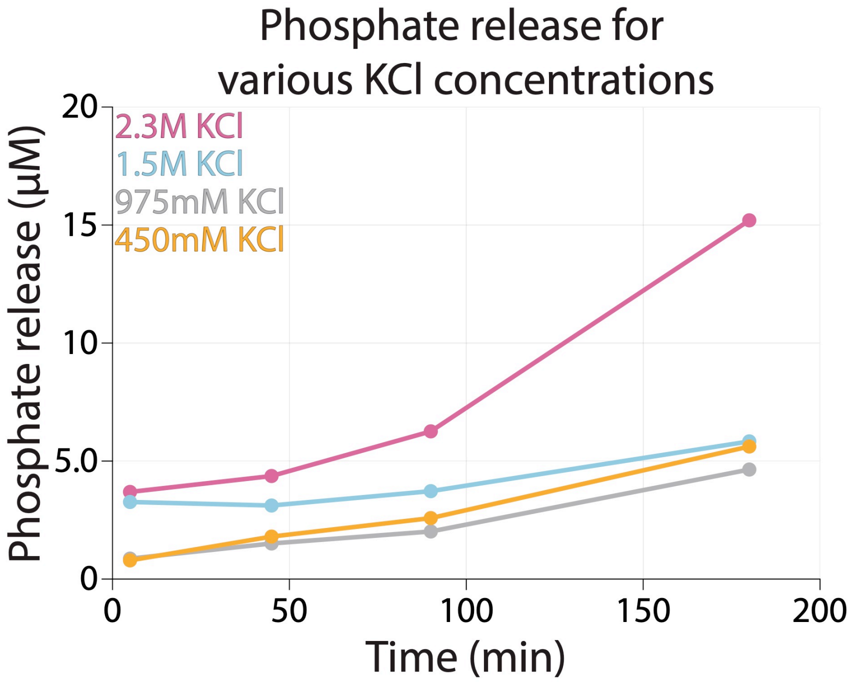

Supplemental Figure S6. Malachite Green Assay using 4 μM Salactin in different salt conditions (450 mM, 975 mM, 1.5 M, 2.29 M KCl). As noted in the main text, the higher ATPase activity suggests polymerization is favored at higher salt concentrations.

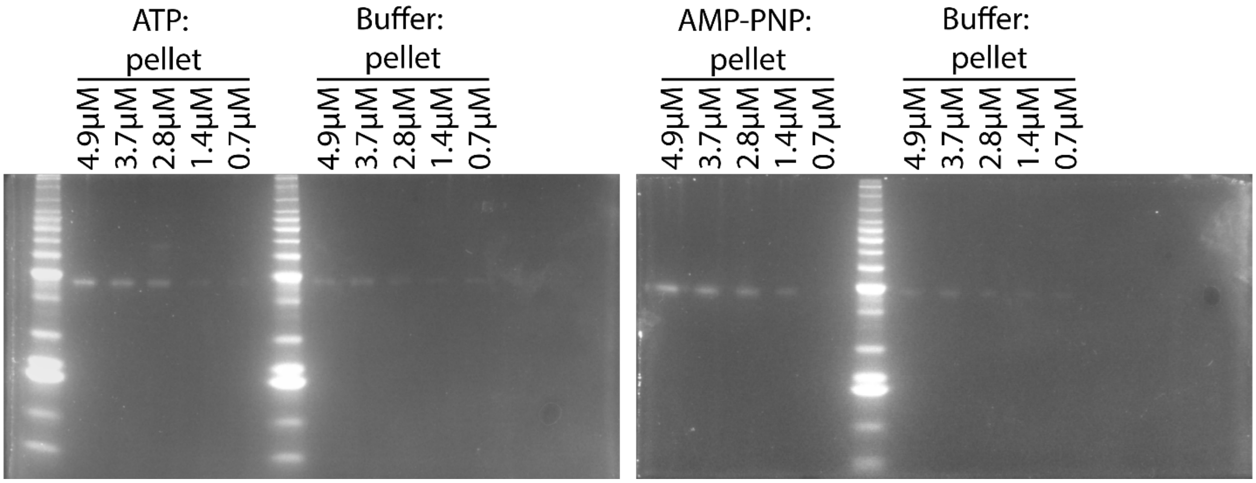

Supplemental Figure S7. Representative pelleting SDS-PAGE gels of Salactin in ATP (*left*) and AMPPNP (*right*) across different protein concentrations. All gels are stained with SYPRO Orange.

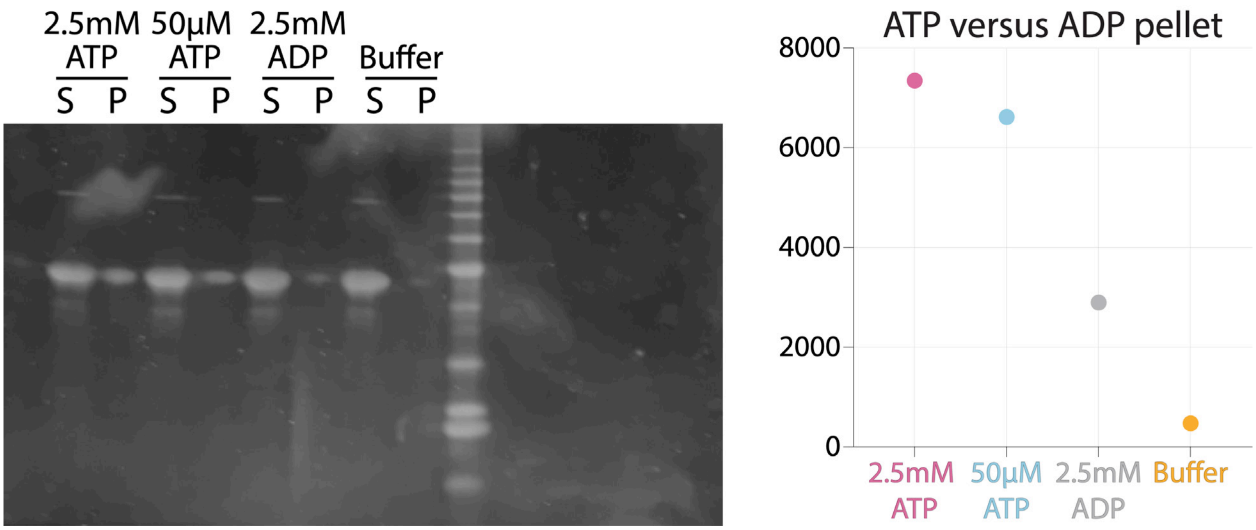

Supplemental Figure S8. Pelleting of Salactin in ATP compared to ADP. SDS-Page gel of 10 µM Salactin in 2.5 mM ATP, 50 µM ATP, 2.5 mM ADP, and HP buffer (*left*). Measured SYPRO Orange intensity of the four different conditions (*right*). All gels are stained with SYPRO Orange. S = supernatant, P = pellet.

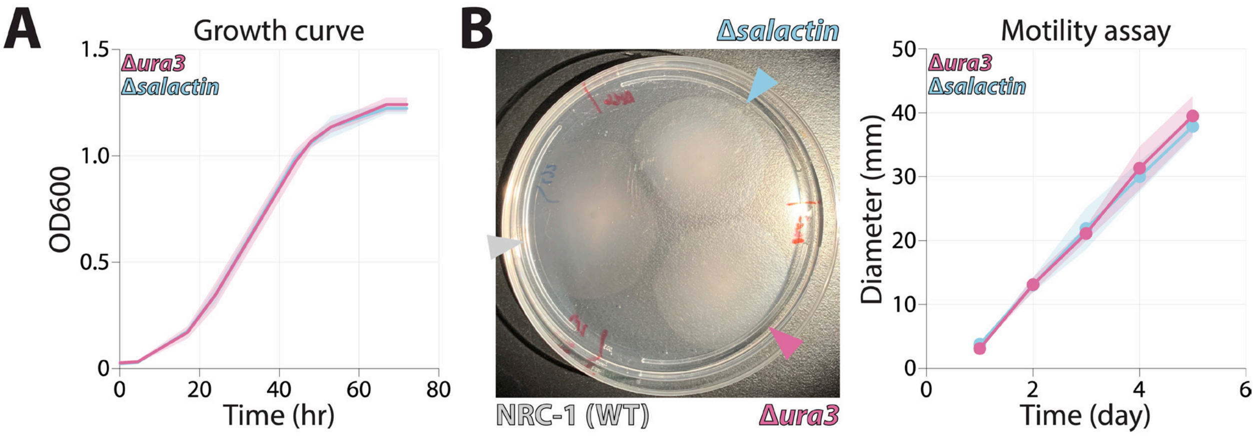

Supplemental Figure S9. (A) Growth curves of Δ*ura3* and Δ*salactin* cells in rich media. Data is from 6 biological replicates. (B) Representative image of Δ*ura3* and Δ*salactin* on an agar motility plate (10% CM, 0.3% agar) 5 days after inoculation *(left*). Δ*ura3* is in the bottom right corner, Δ*salactin* is in the upper right corner of the plate, and NRC-1 (wild-type cells) are on the left. Quantitation of 9 plates (N = 9) across 5 days indicated no motility defect in the Δ*salactin* strain relative to the Δ*ura3* strain (p-value >0.05 for each of the five days) (*right*).

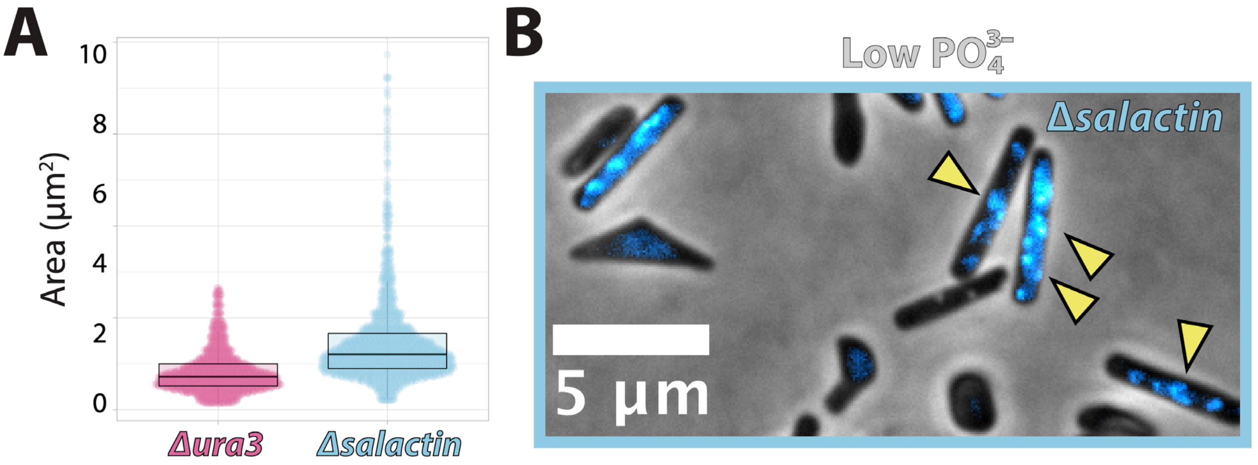

Supplemental Figure S10. (A) Area of Δ*ura3* and Δ*salactin* cells in standard phosphate media at stationary phase. N = 1337 for Δ*ura3* and 1506 for Δ*salactin.* (B) Zoomed-in image of Δ*salactin* cells in low phosphate demonstrating that cells now show clear foci of DNA as indicated by the yellow arrowheads.

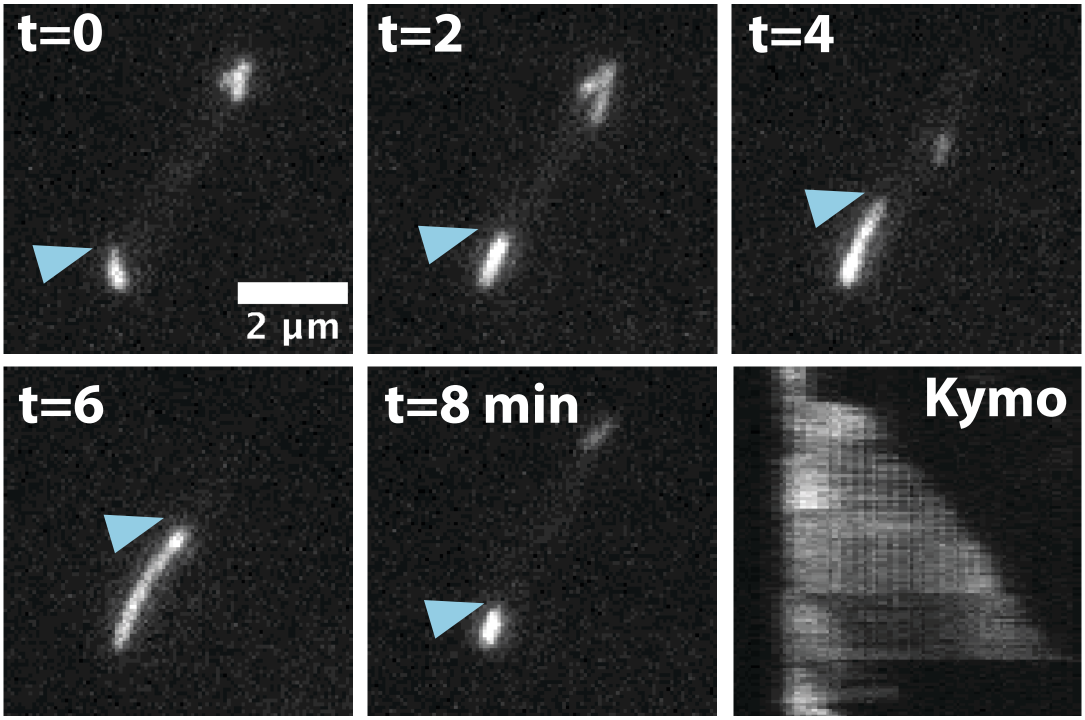

Supplemental Figure S11 – Montage of Salactin-msfGFP (strain hsJZ52) filament dynamics in low phosphate media. Images are on the same scale and the scale bar on the first panel (2 μm) applies to all panels.

SUPPLEMENTAL MOVIES

SM1. Video of Salactin-msfGFP expressed on top of the native copy, visualized by Near-TIRF fluorescent microscopy. Images were taken with a 488nm laser every 30 seconds for 1 hour. Video is 600x actual speed. Scale bar = 4 µm. Movie corresponds to the montage in Figure 2A.

SM2. Video of Salactin-HaloTag expressed on top of the native copy visualized by Near-TIRF fluorescent microscopy. Images were taken every 10 seconds for 20 minutes. Video is 150x actual speed. Cyan is Salactin-HaloTag labeled with low concentrations of JF549 to generate speckles, and magenta is Salactin-HaloTag labeled with high concentrations of JF505 to label the whole filament. Scale bar = 2 µm. Movie corresponds to the montage in Figure 2F.

SM3. Video of Salactin-HaloTag labeled with JF549 that was expressed on top of the native copy, visualized by Near-TIRF fluorescent microscopy Images were taken every 30 seconds for 30 minutes. Video is 600x actual speed. Scale bar = 4 µm.

SM4. Video of Salactin-HaloTag expressed as a sole copy visualized by Near-TIRF fluorescent microscopy. Images were taken every 30 seconds for 1 hour. Video is 600x actual speed. Scale bar = 4 µm.

SM5. Video of Salactin-msfGFP expressed as a sole copy visualized by Near-TIRF fluorescent microscopy. Images were taken every 2 minutes for 32 minutes. Video is 1,200x actual speed. Scale bar = 10 µm.

SM6. Video of Salactin-msfGFP expressed on top of the native copy (hsJZ52). Cells were grown in low phosphate and visualized by Near-TIRF fluorescent microscopy. Images were taken every 5 seconds for 10 minutes. Video is 20x actual speed.

SUPPLEMENTAL TABLES

Supplemental Table S1. Percent identity of Salactin to other polymerizing actin fold proteins.

Protein	% identity to Salactin	PDB ID	RMSD
C. crescentus MreB	21.8%	4CZL	2.386
S. cerevisiae actin	10.5%	1YAG	2.881
M. magneticum MamK	23.0%	5LJV	2.573
R1 ParM	8.2%	1MWM	5.182
B. subtilis Alp7A	17.1%	5EC0	2.913
B. subtilis AlfA	13.8%	6BQW	5.505
P. calidifontis Crenactin	19.7%	5MW1	2.702

Supplemental Table S2. Distribution of cells with filaments that are dynamic or not dynamic.

	WT (*prpa-salactin-msfGFP*) (n = 334)	WT (*prpa-salactin-halotag*) (n=170)	Δ*salactin* (*prpa-salactin-halotag*) (n=129)
Dynamic filaments	74.55%	61.18%	38.76%
No dynamics	25.45%	38.82%	61.24%

Supplemental Table S3. The distribution of the different phenotypes in Δ*salactin* (*prpa-salactin-msfGFP*): split between foci, diffuse, and filaments.

	Δ*salactin* (*prpa-salactin-msfGFP*) (n = 1061)
Foci	93.2%
Diffuse	4.4%
Filaments	2.4%

Supplemental Table S4. Strains used in this study.

Strain	Genotype	Source
HS148	Δ*ura3* (*H. salinarum*)	(3)
ΔVNG0153C	Δ*salactin* (*H. salinarum*)	Gift from Baliga lab
hsJZ52	WT (*prpa-salactin-msfGFP*) (*H. salinarum*)	This study
hsJZ86	WT (*prpa-salactin-halotag*) (*H. salinarum*)	This study
hsJZ95	Δ*salactin* (*prpa-salactin-msfGFP*) (*H. salinarum*)	This study
hsJZ106	Δ*salactin* (*prpa-salactin-halotag*) (*H. salinarum*)	This study
HsJZ118	Δ*salactin* (*Psal-salactin*) (*H. salinarum*)	This study

Supplemental Table S5. Plasmids used in this study.

Plasmid	Description	Source
pHS01	pRPA plasmid	This study
pJZHS4	*prpa-salactin-15aa-msfGFP*	This study
pJZHS11	*prpa-salactin-15aa-halotag*	This study
pEJZ2	*pSUMO-salactin*	This study
pEJZ15	*pSUMO-salactin-GSKCK*	This study
pHSJZ22	*Psalactin-salactin*	This study

Supplemental Table S6. Primers used in this study.

Primer	Sequence	Description	Source
oJM220	TCGACGATGTCGATGGTGAC	Used to create qPCR standard curves	This study
oJM221	AGCAAGGATGGGACTGTTCG	Used to create qPCR standard curves	This study
oPS48	CTCGAGCCCGGGTG	Used to amplify linear pSUMO backbone	(4)
oPS49	ACCACCAATCTGTTCTCTGTG	Used to amplify linear pSUMO backbone	(4)
oHS01	CGGGTACGCCGAAAGCTTGGAT CCGAATTCTGTCGGTTCAGGCCA AGG	Used to amplify rpa promoter (reverse) with a PMTFChis backbone tail (for pHS01)	This study
oHS02	CCCTCCCATGCCACTCTTCACAC GCGGTACCTGGCTCCGCAAGCC AAG	Used to amplify rpa promoter (forward) with a PMTFChis backbone tail (for pHS01)	This study
oHS263	GGCCTGAGCCCGGTCCCTGGCC AGATCCCTCGAGGTACTCCCCGA GATCGA	Used to amplify *salactin* (reverse) with a part of the 15 amino acid linker tail (for pJZHS4 and pJZHS11)	This study
oJZHs28	GCCTTGGCCTGAACCGACAGAT GTCCGACGATACCGAG	Used to amplify *salactin* (forward) with a rpa promoter tail (for pJZHS4 and pJZHS11)	This study
oJZHs44	GACCGGGCTCAGGCCAAGGTTC CGGCCGAAAAGGGGAAGAATTG	Used to amplify msfGFP (forward) with a part of the 15 amino acid linker tail (for pJZHS4)	This study
oJZHs48	CCGAAAGCTTGGATCCGCTATCA TTTGTAAAGTTCATCCATTC	Used to amplify msfGFP (reverse) with a prpa backbone tail (for pJZHS4)	This study
oJZHs57	CAGAGAACAGATTGGTGGTATG TCCGACGATACCG	Used to amplify *salactin* (forward) with a pSUMO backbone tail (for pEJZ2 and pEJZ15)	This study
oJZHs58	CCCGGGCTCGAGCTAGTACTCCC CGAGATC	Used to amplify *salactin* (reverse) with a pSUMO backbone tail (for pEJZ2)	This study

oJZHs87	CGGGCTCAGGCCAAGGTTCCGG CGCAGAAATCGGTACTGGC	Used to amplify HaloTag (forward) with a part of the 15 amino acid linker tail (for pJZHS11)	This study
oJZHs88	GAAAGCTTGGATCCGCTATTAGC CGCTGATTTCTAAGGT	Used to amplify Halotag (reverse) with a prpa backbone tail (for pJZHS11)	This study
oJZHs118	CACCCGGGCTCGAGCTATTTGCA TTTGCTGCCGTACTCCCCGAGAT CG	Used to amplify *salactin* (reverse) with a GSKCK+pSUMO backbone tail (for pEJZ15)	This study
oJZHs127	TGCCACTCTTCACACGCGGTACC AAAGAAGTGCTGTCGGC	Used to amplify the natural promoter region of salactin (forward) together with salactin gene (for pHSJZ22)	This study
oJZHs90	GGCCCATTCAACGGGCCTACAT GTCCGACGATACCGA	Used to amplify the natural promoter region of salactin together with salactin gene (for pHSJZ22)	This study

SUPPLEMENTAL FILES

Supplemental files are available in the Dataverse repository at https://doi.org/10.7910/DVN/JPN2C4

Supplemental File S1 Contains alignments (.faa), newicks (.treefile), and renamed newicks (.renamed) for each of the phylogenetic analyses; in addition to that, we also include the hmm profiles (.hmm), the results for the hmm search (.hmmtbl), and unaligned sequences from these results (.hmmered_fasta).

Supplemental File S2. Python session file containing a backbone alignment of the published structures of MreB, MamK, actin, ParM, Alfa, Alp7A, and crenactin to the predicted Alphafold structure of Salactin.

Supplemental File S3.
Excel sheet containing whole genome resequencing data for Δ*salactin*, Δ*ura3*, and NRC-1 wildtype (as a baseline for genomic differences). Each page represents differences from the NRC-1 genome with the specified strain.

SUPPLEMENTAL METHODS – PLASMID CONSTRUCTION

pHS01: Modified pMTFChis (Darnell et al., 2020), with a rpa (gene locus tag: VNG0133G (old), VNG_RS00545) promoter instead of a fdx (gene locus tag: VNG2293G (old), VNG_RS00545) promoter was generated with two fragments: 1) the RPA promoter (with pMTFChis backbone overhang) was PCR amplified using oHS01 and oHS02 from the NRC-1 *H. salinarum* genome, 2) the plasmid backbone created by cutting the pMTFChis plasmid with KpnI and EcoRI restriction enzymes. The two pieces were assembled using Gibson assembly (5)

pJZHS4: *prpa-salactin-15aa-msfGFP* was generated with three fragments: 1) *salactin* (with plasmid backbone and 15 amino acid linker (15aa) overhang), which was PCR amplified using oHS263 and oJZHs28 from the NRC-1 genome, 2) msfGFP (with plasmid backbone and 15aa overhang), which was PCR amplified using oJZHs44 and oJZHs48 from a gBlock gene fragment obtained from Dion and colleagues (6). 3) linear pHS01 backbone, which was made with restriction enzyme digest with EcoRI. The three pieces were assembled using Gibson assembly.

pJZHS11: *prpa-salactin-15aa-halotag* was generated with three fragments: 1) *salactin*, which was PCR amplified using oHS263 and oJZHs28 from the NRC-1 genome or pJZHS4, 2) HaloTag, which was PCR amplified using oJZHs87 and oJZHs88 from a gBlock gene fragment obtained from Dion and colleagues (6), 3) linear pHS01 backbone, which was made with restriction enzyme digest with EcoRI. The three pieces were assembled using Gibson assembly.

pEJZ2: *pSUMO-salactin* (his6-SUMO tagged Salactin in a T7 expression vector) was generated with two fragments: 1) *salactin* (with pSUMO backbone overhang), which was PCR amplified using oJZHs57 and

oJZHs58 from the NRC-1 genome or pJZHS4, 2) linear pSUMO backbone, which was PCR amplified using oPS48 and oPS49. The two pieces were assembled using Gibson assembly.

pEJZ15: *pSUMO-salactin-GSKCK* was generated with two fragments: 1) *salactin* (with pSUMO backbone overhang and addition of the GSKCK), which was PCR amplified using oJZHs57 and oJZHs118 from the NRC-1 genome or pJZHS4, 2) linear pSUMO backbone, which was PCR amplified using oPS48 and oPS49. The two pieces were assembled using Gibson assembly.

pHSJZ22: *pSalactin-salactin* was generated with two fragments: 1) *salactin* promoter region together with salactin's ORF, which was PCR amplified using oJZHs127 and oJZHs90 from the NRC-1 genome 4, 2) linear pHS01 backbone, which was made with restriction enzyme digest with KpnI and EcoRI. The two pieces were assembled using Gibson assembly.

SUPPLEMENTAL REFERENCES

1. Minh BQ, Schmidt HA, Chernomor O, Schrempf D, Woodhams MD, Haeseler A von, Lanfear R. 2020. IQ-TREE 2: New models and efficient methods for phylogenetic inference in the genomic era. Mol Biol Evol 37:1530–1534.
2. Hoang DT, Chernomor O, Haeseler A von, Minh BQ, Vinh LS. 2018. UFBoot2: Improving the Ultrafast Bootstrap Approximation. Mol Biol Evol 35:518–522.
3. Darnell CL, Zheng J, Wilson S, Bertoli RM, Bisson-Filho AW, Garner EC, Schmid AK. 2020. The Ribbon-Helix-Helix Domain Protein CdrS Regulates the Tubulin Homolog ftsZ2 To Control Cell Division in Archaea. mBio 11:e01007-20.
4. Stoddard PR, Lynch EM, Farrell DP, Dosey AM, DiMaio F, Williams TA, Kollman JM, Murray AW, Garner EC. 2020. Polymerization in the actin ATPase clan regulates hexokinase activity in yeast. Science 367:1039–1042.
5. Gibson DG, Young L, Chuang RY, Venter JC, Hutchison CA, Smith HO. 2009. Enzymatic assembly of DNA molecules up to several hundred kilobases. Nature Methods 6:343-U41.
6. Dion MF, Kapoor M, Sun Y, Wilson S, Ryan J, Vigouroux A, Teeffelen S van, Oldenbourg R, Garner EC. 2019. Bacillus subtilis cell diameter is determined by the opposing actions of two distinct cell wall synthetic systems. Nat Microbiol 4:1294–1305.
